# Supplementary material for: Membrane curvature regulates the spatial distribution of bulky glycoproteins
Source: Nat Commun. 2022 Jun 2;13:3093. doi: 10.1038/s41467-022-30610-2 (PMC9163104; doi:10.1038/s41467-022-30610-2)
Supplement: Supplementary file 1 — Supplementary Information [file 41467_2022_30610_MOESM1_ESM.pdf]

Supplemental Information for

Membrane curvature regulates the spatial distribution of bulky glycoproteins

Chih-Hao Lu[a], Kayvon Pedram[a,b], Ching-Ting Tsai[a], Taylor Jones IV[a], Xiao Li[a,c], Melissa Nakamoto[a], Carolyn R. Bertozzi[a,d,e], and Bianxiao Cui[a,*]

[a]Department of Chemistry, Stanford University, Stanford, CA, USA, 94305
[b]Janelia Research Campus, Howard Hughes Medical Institute, VA, USA, 20147 (Current address)
[C]School of Mechanical Engineering, Xi'an Jiaotong University, Xi'an, China (Current address)
[d]Stanford ChEM-H, Stanford University, Stanford, CA, USA, 94305
[e]Howard Hughes Medical Institute, Stanford University, Stanford, USA, CA 94305

[*] To whom should be addressed: Bianxiao Cui, E-mail: bcui@stanford.edu

Target	Normalized nanobar end-to-side ratio - **U2OS** cells on **200-nm nanobars**					
	Mean	SD	SEM (SD/$\sqrt{N}$)	N (# cells)	n (# nanobars)	Corresponding figure
α-AP2	2.043	0.647	0.138	22	290	Fig. 1J
F-actin	1.384	0.371	0.085	19	157	
CAAX	1.000	0.038	0.003	170	9092	
MUC1ΔCT-0TR	1.028	0.047	0.008	38	1757	
MUC1ΔCT-10TR	0.999	0.042	0.006	50	2097	
MUC1ΔCT-21TR	0.900	0.047	0.007	48	2202	
MUC1ΔCT-42TR	0.889	0.043	0.005	63	3415	

Supplementary Table 1. Statistical analysis of normalized nanobar end-to-side ratios on 200-nm nanobar arrays for U2OS cells.

A

Target	Normalized pillar-to-cytosolic background ratio - **HeLa** cells on **200-nm nanopillars**					
	Mean	SD	SEM (SD/$\sqrt{N}$)	N (# cells)	n (# nanopillars)	Corresponding figure
α-MUC1	0.899	0.188	0.036	27	2705	Fig. 1N & Suppl. Fig. 4C
CellMask	1.000	0.158	0.032	25	3647	
α-AP2	1.624	0.315	0.067	22	1734	Fig. 1N
F-actin	1.250	0.159	0.046	12	2943	Suppl. Fig. 4C

B

Target	Normalized pillar-to-cytosolic background ratio - **U2OS** cells on **200-nm nanopillars**					
	Mean	SD	SEM (SD/$\sqrt{N}$)	N (# cells)	n (# nanopillars)	Corresponding figure
MUC1ΔCT-42TR	0.807	0.102	0.025	16	1576	Fig. 1O, 4G, 4I & Suppl. Fig. 5E
MUC1ΔCT-0TR	1.148	0.209	0.044	23	1853	Fig. 1O
CAAX	1.000	0.196	0.026	59	5720	Fig. 1O, 4G, 4I & Suppl. Fig. 5E
MUC1ΔCT-42TR mutant	0.909	0.141	0.039	13	728	Fig. 4G
MUC1ΔCT-42TR +StcE	0.978	0.2054	0.044	22	2361	Fig. 4I
α-AP2	1.351	0.175	0.044	16	1735	Fig. 1O
F-actin	1.228	0.181	0.047	15	1252	Suppl. Fig. 5E

Supplementary Table 2. Statistical analysis of normalized pillar-to-cytosolic background ratios on 200-nm nanopillar arrays. (A) HeLa cells; **(B)** U2OS cells.

A

Target	Inner angle of NanoX (deg)	Type of ratio (Type of curvature)	Normalized intensity ratio - U2OS cells on **gradient nanoXs**					
			Mean	SD	SEM (SD/$\sqrt{N}$)	N (# cells)	n (# nanoXs)	Corresponding figure
MUC1 ΔCT-42TR	30	End-to-side (+)	0.885	0.064	0.017	15	77	Fig. 2F
		Inner-to-side_1 (-)	0.986	0.112	0.029			
		Inner-to-side_2 (-)	0.978	0.154	0.040			
	45	End-to-side (+)	0.910	0.117	0.029	16	78	Not shown
		Inner-to-side_1 (-)	1.083	0.194	0.048			
		Inner-to-side_2 (-)	1.040	0.088	0.022			
	60	End-to-side (+)	0.921	0.124	0.034	13	80	Fig. 2F
		Inner-to-side_1 (-)	1.072	0.131	0.036			
		Inner-to-side_2 (-)	1.107	0.193	0.053			
	75	End-to-side (+)	0.869	0.111	0.032	12	68	Not shown
		Inner-to-side_1 (-)	1.104	0.179	0.052			
		Inner-to-side_2 (-)	1.030	0.129	0.037			
	90	End-to-side (+)	0.821	0.138	0.038	13	70	Fig. 2F
		Inner-to-side_1 (-)	1.076	0.129	0.036			
		Inner-to-side_2 (-)	1.079	0.128	0.035			

B

Target	Inner angle of NanoX (deg)	Type of ratio (Type of curvature)	Normalized intensity ratio - U2OS cells on **gradient nanoXs**					
			Mean	SD	SEM (SD/$\sqrt{N}$)	N (# cells)	n (# nanoXs)	Corresponding figure
MUC1 ΔCT-0TR	30	End-to-side (+)	0.978	0.064	0.016	17	89	Suppl. Fig. 8A
		Inner-to-side_1 (-)	0.975	0.064	0.016			
		Inner-to-side_2 (-)	0.998	0.108	0.026			
	45	End-to-side (+)	0.985	0.118	0.026	20	103	Not shown
		Inner-to-side_1 (-)	1.066	0.111	0.025			
		Inner-to-side_2 (-)	1.058	0.095	0.021			
	60	End-to-side (+)	0.992	0.099	0.025	16	84	Suppl. Fig. 8A
		Inner-to-side_1 (-)	1.018	0.107	0.027			
		Inner-to-side_2 (-)	0.971	0.098	0.024			
	75	End-to-side (+)	0.974	0.101	0.038	7	34	Not shown
		Inner-to-side_1 (-)	1.023	0.056	0.021			
		Inner-to-side_2 (-)	0.944	0.066	0.025			
	90	End-to-side (+)	1.047	0.071	0.029	6	29	Suppl. Fig. 8A
		Inner-to-side_1 (-)	1.007	0.054	0.022			
		Inner-to-side_2 (-)	1.028	0.109	0.044			

C

Target	Inner angle of NanoX (deg)	Type of ratio (Type of curvature)	Normalized intensity ratio - **U2OS** cells on **gradient nanoXs**					
			Mean	SD	SEM ($SD/\sqrt{N}$)	N (# cells)	n (# nanoXs)	Corresponding figure
F-actin	30	End-to-side (+)	1.164	0.209	0.036	34	232	Fig. 2G
		Inner-to-side_1 (-)	0.882	0.125	0.021			
		Inner-to-side_2 (-)	0.969	0.155	0.027			
	45	End-to-side (+)	1.207	0.214	0.036	36	237	Not shown
		Inner-to-side_1 (-)	0.915	0.147	0.024			
		Inner-to-side_2 (-)	0.926	0.137	0.023			
	60	End-to-side (+)	1.315	0.287	0.047	37	240	Fig. 2G
		Inner-to-side_1 (-)	0.981	0.143	0.024			
		Inner-to-side_2 (-)	0.979	0.096	0.016			
	75	End-to-side (+)	1.329	0.398	0.078	26	174	Not shown
		Inner-to-side_1 (-)	1.012	0.135	0.026			
		Inner-to-side_2 (-)	1.015	0.137	0.027			
	90	End-to-side (+)	1.266	0.271	0.053	26	148	Fig. 2G
		Inner-to-side_1 (-)	0.950	0.169	0.033			
		Inner-to-side_2 (-)	0.961	0.172	0.034			

Supplementary Table 3. Statistical analysis of normalized intensity ratios on gradient nanoX arrays for U2OS cells. (A) MUC1-ΔCT_42TR-GFP; **(B)** MUC1-ΔCT_0TR-GFP; **(C)** F-actin.

A

Target	Colocalization analysis (Pearson's correlation coefficient) with **IRSp53** - **U2OS** cells				
	Mean	SD	SEM (SD/$\sqrt{N}$)	N (# cells)	Corresponding figure
MUC1ΔCT-0TR	0.509	0.118	0.024	24	Fig. 3E
MUC1ΔCT-10TR	0.475	0.124	0.030	17	Fig. 3E & 4E
MUC1ΔCT-21TR	0.506	0.127	0.028	20	
MUC1ΔCT-42TR	0.418	0.089	0.022	16	
MUC1ΔCT-10TR mutant	0.490	0.133	0.050	7	Fig. 4E
MUC1ΔCT-21TR mutant	0.437	0.127	0.042	9	
MUC1ΔCT-42TR mutant	0.478	0.166	0.033	25	

B

Target	Colocalization analysis (Pearson's correlation coefficient) with **FBP17** - **U2OS** cells				
	Mean	SD	SEM (SD/$\sqrt{N}$)	N (# cells)	Corresponding figure
MUC1ΔCT-0TR	0.455	0.099	0.021	22	Fig. 3E
MUC1ΔCT-10TR	0.316	0.139	0.025	30	Fig. 3E & 4F
MUC1ΔCT-21TR	0.221	0.096	0.019	25	
MUC1ΔCT-42TR	0.252	0.086	0.017	27	
MUC1ΔCT-10TR mutant	0.382	0.107	0.020	28	Fig. 4F
MUC1ΔCT-21TR mutant	0.417	0.113	0.025	20	
MUC1ΔCT-42TR mutant	0.340	0.122	0.021	33	

C

Target	Colocalization analysis (Pearson's correlation coefficient) with α-MUC1 - **HeLa** cells				
	Mean	SD	SEM (SD/$\sqrt{N}$)	N (# cells)	Corresponding figure
IRSp53	0.250	0.074	0.030	6	Fig. 3F
FBP17	0.115	0.049	0.013	14	

D

Target	Colocalization analysis (Pearson's correlation coefficient) with MUC1(FL) – **U2OS** cells				
	Mean	SD	SEM (SD/$\sqrt{N}$)	N (# cells)	Corresponding figure
IRSp53	0.535	0.101	0.020	26	Suppl. Fig. 12C
FBP17	0.343	0.085	0.014	35	

Supplementary Table 4. Statistical analysis of degree of colocalization (Pearson's correlation coefficient). (A) between IRSp53-mCherry and 7 different MUC1-ΔCT-GFP in U2OS cells; **(B)** between mCherry-FBP17 and 7 different MUC1-ΔCT-GFP in U2OS cells; **(C)** between IRSp53-mCherry or mCherry-FBP17 with α-MUC1 in HeLa cells; **(D)** between IRSp53-mCherry or mCherry-FBP17 with full-length MUC1-GFP in U2OS cells.

A

Target	Inner angle of NanoX (deg)	Type of ratio (Type of curvature)	Normalized intensity ratio – **SLB** with **30% DGS-Ni-NTA** on **gradient nanoXs**					
			Mean	SD	SEM ($SD/\sqrt{N}$)	N (# Fields of view)	n (# nanoXs)	Corresponding figure
Podxl	30	End-to-side (-)	1.143	0.109	0.026	18	280	Fig. 5D
		Inner-to-side_1 (+)	0.896	0.055	0.013			
		Inner-to-side_2 (+)	0.918	0.067	0.018			
	45	End-to-side (-)	1.115	0.088	0.020	19	288	Not shown
		Inner-to-side_1 (+)	0.874	0.062	0.014			
		Inner-to-side_2 (+)	0.899	0.093	0.021			
	60	End-to-side (-)	1.122	0.093	0.021	19	302	Fig. 5D
		Inner-to-side_1 (+)	0.874	0.061	0.014			
		Inner-to-side_2 (+)	0.848	0.083	0.019			
	75	End-to-side (-)	1.106	0.107	0.025	19	307	Not shown
		Inner-to-side_1 (+)	0.897	0.075	0.017			
		Inner-to-side_2 (+)	0.864	0.081	0.019			
	90	End-to-side (-)	1.111	0.112	0.026	18	263	Fig. 5D
		Inner-to-side_1 (+)	0.858	0.069	0.016			
		Inner-to-side_2 (+)	0.895	0.071	0.017			

B

Target	Inner angle of NanoX (deg)	Type of ratio (Type of curvature)	Normalized intensity ratio – **SLB** with **30% DGS-Ni-NTA** on **gradient nanoXs**					
			Mean	SD	SEM ($SD/\sqrt{N}$)	N (# Fields of view)	n (# nanoXs)	Corresponding figure
Degly. Podxl	30	End-to-side (-)	1.009	0.080	0.021	15	210	Fig. 5E
		Inner-to-side_1 (+)	1.054	0.172	0.044			
		Inner-to-side_2 (+)	1.009	0.081	0.021			
	45	End-to-side (-)	1.007	0.096	0.025	15	245	Not shown
		Inner-to-side_1 (+)	1.023	0.145	0.037			
		Inner-to-side_2 (+)	1.003	0.082	0.021			
	60	End-to-side (-)	0.971	0.086	0.022	15	244	Fig. 5E
		Inner-to-side_1 (+)	1.005	0.111	0.022			
		Inner-to-side_2 (+)	1.005	0.086	0.020			
	75	End-to-side (-)	0.975	0.135	0.035	15	246	Not shown
		Inner-to-side_1 (+)	1.020	0.122	0.031			
		Inner-to-side_2 (+)	0.992	0.095	0.024			
	90	End-to-side (-)	0.961	0.078	0.020	15	247	Fig. 5E
		Inner-to-side_1 (+)	0.985	0.164	0.042			
		Inner-to-side_2 (+)	0.994	0.157	0.040			

C

Target	Inner angle of NanoX (deg)	Type of ratio (Type of curvature)	Normalized intensity ratio – **SLB** with **10% DGS-Ni-NTA** on **gradient nanoXs**					
			Mean	SD	SEM (SD/$\sqrt{N}$)	N (# Fields of view)	n (# nanoXs)	Corresponding figure
Podxl	30	End-to-side (-)	1.073	0.059	0.018	11	156	Fig. 5G
		Inner-to-side_1 (+)	1.148	0.180	0.054			
		Inner-to-side_2 (+)	1.010	0.102	0.031			
	45	End-to-side (-)	1.085	0.057	0.017	11	174	Not shown
		Inner-to-side_1 (+)	1.191	0.156	0.047			
		Inner-to-side_2 (+)	1.021	0.080	0.024			
	60	End-to-side (-)	1.092	0.095	0.029	11	184	Fig. 5G
		Inner-to-side_1 (+)	1.111	0.130	0.039			
		Inner-to-side_2 (+)	0.996	0.065	0.020			
	75	End-to-side (-)	1.082	0.149	0.045	11	176	Not shown
		Inner-to-side_1 (+)	1.153	0.106	0.032			
		Inner-to-side_2 (+)	1.050	0.074	0.022			
	90	End-to-side (-)	1.049	0.060	0.018	11	180	Fig. 5G
		Inner-to-side_1 (+)	1.102	0.093	0.028			
		Inner-to-side_2 (+)	1.133	0.082	0.025			

D

Target	Inner angle of NanoX (deg)	Type of ratio (Type of curvature)	Normalized intensity ratio – **SLB** with **10% DGS-Ni-NTA** on **gradient nanoXs**					
			Mean	SD	SEM (SD/$\sqrt{N}$)	N (# Fields of view)	n (# nanoXs)	Corresponding figure
Degly. Podxl	30	End-to-side (-)	1.093	0.077	0.026	9	125	Fig. 5H
		Inner-to-side_1 (+)	1.065	0.166	0.055			
		Inner-to-side_2 (+)	1.021	0.055	0.018			
	45	End-to-side (-)	1.076	0.061	0.020	9	125	Not shown
		Inner-to-side_1 (+)	1.061	0.095	0.032			
		Inner-to-side_2 (+)	0.977	0.070	0.023			
	60	End-to-side (-)	1.079	0.080	0.027	9	130	Fig. 5H
		Inner-to-side_1 (+)	0.999	0.125	0.042			
		Inner-to-side_2 (+)	0.966	0.077	0.026			
	75	End-to-side (-)	1.115	0.061	0.020	9	145	Not shown
		Inner-to-side_1 (+)	0.983	0.133	0.044			
		Inner-to-side_2 (+)	0.951	0.111	0.037			
	90	End-to-side (-)	1.063	0.085	0.028	9	147	Fig. 5H
		Inner-to-side_1 (+)	1.009	0.171	0.057			
		Inner-to-side_2 (+)	1.012	0.202	0.067			

Supplementary Table 5. Statistical analysis of normalized intensity ratios on gradient nanoX arrays for the SLB experiments. **(A)** Podocalyxin on 30% DGS-Ni-NTA-doped lipid bilayers; **(B)** Deglycosylated podocalyxin on 30% DGS-Ni-NTA-doped lipid bilayers; **(C)** Podocalyxin on 10% DGS-Ni-NTA-doped lipid bilayers; **(D)** Deglycosylated podocalyxin on 10% DGS-Ni-NTA-doped lipid bilayers.

A

Target	Time (min)	Endocytosis analysis (Anti-α-GFP intensity) - **U2OS** cells on **flat surfaces**				
		Mean	SD	SEM ($SD/\sqrt{N}$)	N (# cells)	Corresponding figure
MUC1ΔCT-0TR	15	182.3	94.8	8.58	122	Fig. 6D
	30	273.5	198.7	19.0	110	
	60	500.1	207.6	22.9	82	
MUC1ΔCT-10TR	15	179.4	69.3	8.34	69	
	30	215.2	111.2	11.4	95	
	60	269.1	133.8	10.9	150	
MUC1ΔCT-42TR	15	166.2	81.7	9.19	79	
	30	132.4	65.7	4.71	195	
	60	153.3	85.6	5.77	220	

B

Target	Time (min)	Endocytosis analysis (Anti-α-GFP intensity) - **U2OS** cells on **200-nm nanopillars**				
		Mean	SD	SEM ($SD/\sqrt{N}$)	N (# cells)	Corresponding figure
MUC1ΔCT-0TR	15	270.3	101.5	13.0	61	Fig. 6E
	30	468.5	247.6	26.0	91	
	60	769.8	335.4	30.8	119	
MUC1ΔCT-10TR	15	200.2	69.6	9.14	58	
	30	320.8	162.7	21.0	60	
	60	432.2	208.5	15.9	171	
MUC1ΔCT-42TR	15	176.2	88.3	9.87	80	
	30	151.5	73.9	5.46	183	
	60	174.2	82.6	6.28	173	

C

Target	Substrate	Endocytosis analysis (Anti-α-GFP intensity) - **U2OS** cells after **30 min**				
		Mean	SD	SEM ($SD/\sqrt{N}$)	N (# cells)	Corresponding figure
MUC1ΔCT-42TR +StcE	Flat	216.2	105.7	5.39	385	Fig. 6F
	Nanopillar	303.1	117.2	9.73	145	
MUC1ΔCT-42TR mutant	Flat	258.9	180.9	11.0	271	
	Nanopillar	280.7	182.8	11.5	254	

Supplementary Table 6. Statistical analysis of MUC1 endocytosis levels in U2OS cells. (A) 3 different MUC1-ΔCT-GFP on flat surfaces; **(B)** 3 different MUC1-ΔCT-GFP on 200-nm nanopillar arrays; **(C)** StcE-treated MUC1_42TR-GFP or MUC1_42TR-GFP triple mutant on either flat surfaces or 200-nm nanopillar arrays.

A

Target	Nanobar width (nm)	Normalized nanobar end-to-side intensity ratio - **U2OS** cells on **gradient nanobars**					
		Mean	SD	SEM (SD/$\sqrt{N}$)	N (# cells)	n (# nanobars)	Corresponding figure
MUC1 ΔCT-0TR	200	1.055	0.091	0.019	23	180	Fig. 7J
	300	1.074	0.108	0.026	17	120	
	400	1.083	0.108	0.019	32	262	
	500	1.042	0.105	0.023	20	171	
	600	1.058	0.120	0.023	27	217	
	700	1.054	0.101	0.022	21	181	
	800	1.060	0.133	0.027	24	189	
	900	1.025	0.115	0.029	16	125	
	1000	1.073	0.138	0.026	28	242	
	1200	1.013	0.105	0.025	17	143	
	1600	1.001	0.054	0.014	16	136	
	2000	1.034	0.104	0.028	14	125	

B

Target	Nanobar width (nm)	Normalized nanobar end-to-side intensity ratio - **U2OS** cells on **gradient nanobars**					
		Mean	SD	SEM (SD/$\sqrt{N}$)	N (# cells)	n (# nanobars)	Corresponding figure
MUC1 ΔCT-10TR	200	1.043	0.109	0.029	14	127	Suppl. Fig. S19A
	300	1.017	0.107	0.048	5	39	
	400	1.054	0.199	0.041	24	221	
	500	1.048	0.128	0.036	13	153	
	600	1.048	0.234	0.046	26	208	
	700	0.987	0.074	0.023	10	109	
	800	1.017	0.107	0.020	29	281	
	900	1.006	0.114	0.031	14	187	
	1000	1.029	0.097	0.018	30	292	
	1200	1.020	0.144	0.032	20	138	
	1600	1.027	0.102	0.025	17	116	
	2000	1.024	0.085	0.027	10	82	

C

Target	Nanobar width (nm)	Normalized nanobar end-to-side intensity ratio - **U2OS** cells on **gradient nanobars**					
		Mean	SD	SEM (SD/$\sqrt{N}$)	N (# cells)	n (# nanobars)	Corresponding figure
MUC1 ΔCT-21TR	200	0.934	0.054	0.013	17	153	Suppl. Fig. S19B
	300	0.936	0.037	0.014	7	51	
	400	0.973	0.068	0.014	24	178	
	500	0.958	0.040	0.016	6	54	
	600	0.958	0.073	0.016	22	148	
	700	0.963	0.076	0.029	7	65	
	800	0.962	0.075	0.016	22	175	
	900	0.963	0.091	0.029	10	96	
	1000	0.947	0.057	0.013	18	137	
	1200	0.976	0.070	0.017	17	117	
	1600	0.996	0.104	0.028	14	105	
	2000	0.985	0.054	0.016	12	85	

D

Target	Nanobar width (nm)	Normalized nanobar end-to-side intensity ratio - **U2OS** cells on **gradient nanobars**					
		Mean	SD	SEM (SD/$\sqrt{N}$)	N (# cells)	n (# nanobars)	Corresponding figure
MUC1 ΔCT-42TR	200	0.895	0.115	0.018	38	407	Fig. 7I
	300	0.945	0.062	0.015	18	165	
	400	0.941	0.131	0.023	34	286	
	500	0.922	0.081	0.023	13	109	
	600	0.916	0.103	0.016	41	341	
	700	0.928	0.065	0.017	14	125	
	800	0.913	0.101	0.018	31	245	
	900	0.932	0.070	0.015	21	177	
	1000	0.945	0.122	0.019	41	325	
	1200	0.946	0.128	0.027	23	210	
	1600	1.006	0.049	0.011	19	171	
	2000	1.010	0.085	0.019	19	166	

E

Target	Nanobar width (nm)	Normalized nanobar end-to-side intensity ratio - **U2OS** cells on **gradient nanobars**					
		Mean	SD	SEM ($SD/\sqrt{N}$)	N (# cells)	n (# nanobars)	Corresponding figure
MUC1 ΔCT-42TR +StcE	200	1.022	0.064	0.015	18	159	Suppl. Fig. S19C
	300	1.042	0.099	0.038	7	71	
	400	1.051	0.070	0.017	17	149	
	500	1.074	0.062	0.025	6	70	
	600	1.045	0.069	0.016	19	152	
	700	1.068	0.091	0.032	8	42	
	800	1.052	0.071	0.016	19	162	
	900	1.043	0.075	0.025	9	69	
	1000	1.041	0.066	0.015	18	146	
	1200	1.032	0.055	0.021	7	64	
	1600	1.024	0.047	0.015	10	86	
	2000	1.027	0.056	0.015	14	138	

F

Target	Nanobar width (nm)	Normalized nanobar end-to-side intensity ratio - **U2OS** cells on **gradient nanobars**					
		Mean	SD	SEM ($SD/\sqrt{N}$)	N (# cells)	n (# nanobars)	Corresponding figure
MUC1 ΔCT-42TR mutant	200	0.986	0.075	0.013	36	235	Suppl. Fig. S19D
	300	1.022	0.118	0.025	23	138	
	400	1.017	0.094	0.015	37	250	
	500	0.991	0.086	0.018	23	129	
	600	0.972	0.072	0.013	29	168	
	700	0.978	0.058	0.014	18	94	
	800	0.964	0.077	0.014	30	170	
	900	0.995	0.089	0.017	26	143	
	1000	1.001	0.085	0.015	33	208	
	1200	0.969	0.025	0.009	8	58	
	1600	1.013	0.067	0.017	16	130	
	2000	1.015	0.075	0.020	14	107	

Supplementary Table 7. Statistical analysis of normalized nanobar end-to-side ratios on gradient nanobar arrays for U2OS cells.

Protein encoded	Type of DNA fragments	PCR Template	DNA primer sequence	PCR product length (bp)
(These three plasmids share the same vector)	Linear Vector	pPB_Muc1_0_mOxGFP_dCT_BlpI	GACCGAGGTGACATCCTGTC	6451
			GCCTCAGGCTCTGCATCAG	
MUC1ΔCT-42TR-mOxGFP Triple Mutant	Insert 1	pPB_Tet_SumoStar_Muc1_21T_rtTAsM2_IRES_NeoR	GCAGGTCTTGCATCAGGGCCTGAGGCAGCAGCCGTA	1304
			CAGGATGTCACCTCGGTCC	
	Insert 2		CCTGATGCAAGACCTGCCCCTGGTGCGACAGCACCA	1278
			TGATGCAGAGCCTGAGGCAGCAGCCGTA	
MUC1ΔCT-21TR-mOxGFP Triple Mutant	Insert		CAGGATGTCACCTCGGTCC	1296
			TGATGCAGAGCCTGAGGCAGCAGCCGTA	
MUC1ΔCT-10TR-mOxGFP Triple Mutant	Insert		CAGGATGTCACCTCGGTCC	636
			TGATGCAGAGCCTGAGGCTGCAGCTGTCACACCATGC	

Supplementary Table 8. DNA sequences of primers used for MUC1ΔCT-mOxGFP Triple Mutant plasmid construction.

The PCR products were then subject to Gibson Assembly. Two PCR templates are kind gifts from Matthew Paszek Lab at Cornell University. All DNA primers were purchased from Integrated DNA Technologies (IDT).

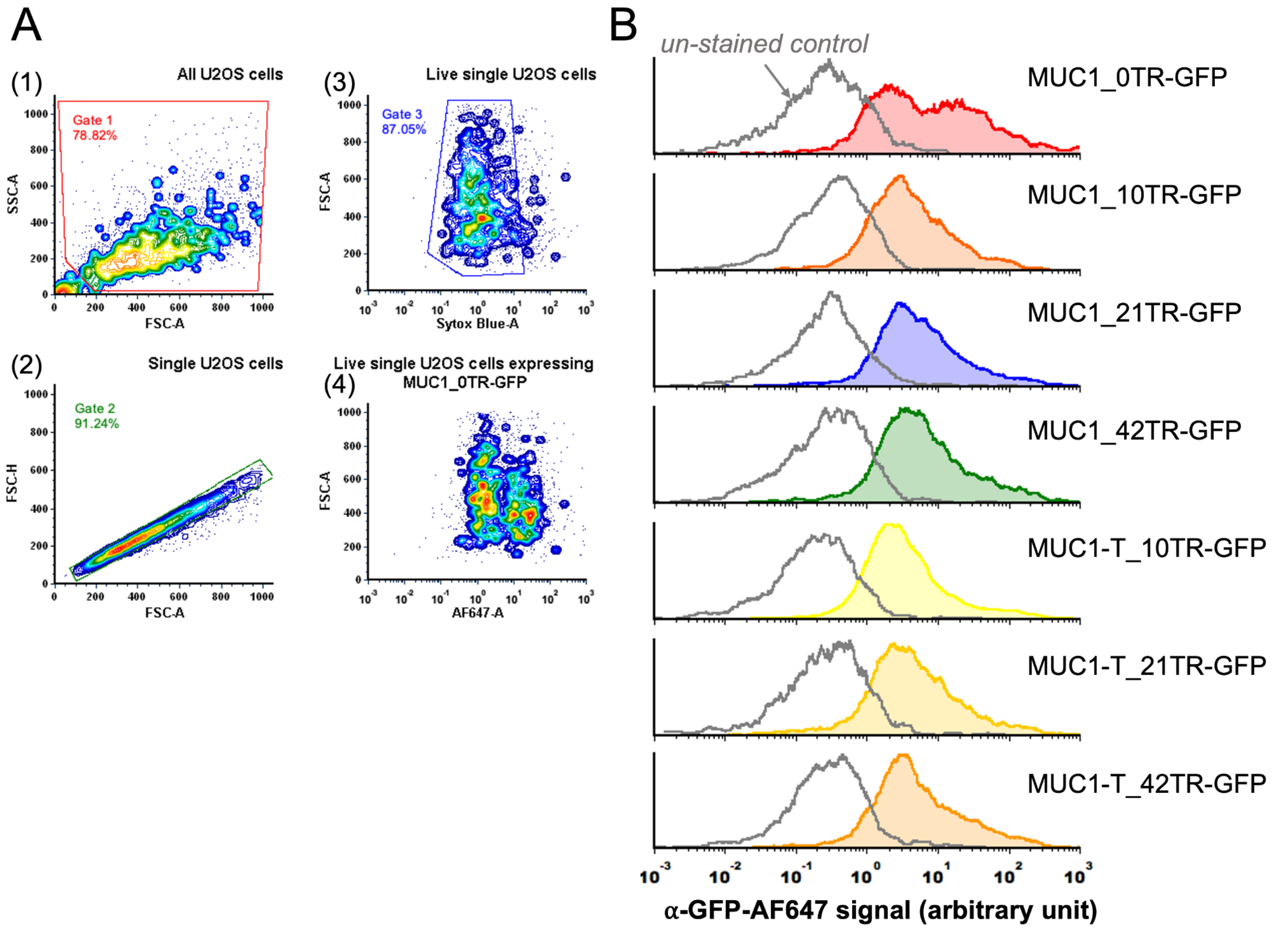

Supplementary Figure 1. Flow cytometric results show comparable cell surface expression levels of various MUC1-ΔCT-GFP in U2OS cells.

(A) Representative flow cytometric gating strategy for determining various MUC1-ΔCT-GFP expressions in U2OS cells. Briefly, (1) Cell debris were first excluded and (2) U2OS cells were then gated for single cells. Subsequently, (3) live single U2OS cells were gated based on Sytox Blue staining. (4) Cell surface expression levels of various MUC1-ΔCT-GFP were then determined. **(B)** Histograms of cell surface expression levels of various MUC1-ΔCT-GFP in U2OS cells. All MUC1-ΔCT-GFP were labeled with rabbit anti-GFP antibodies and goat anti-rabbit IgG Alexa Fluor 647 (AF647). After applying the gating strategy, there are ~3500-18000 cells included per population.

Step 1

mCherry-CAAX channel | MUC1-42TR-GFP channel | Bright Field channel

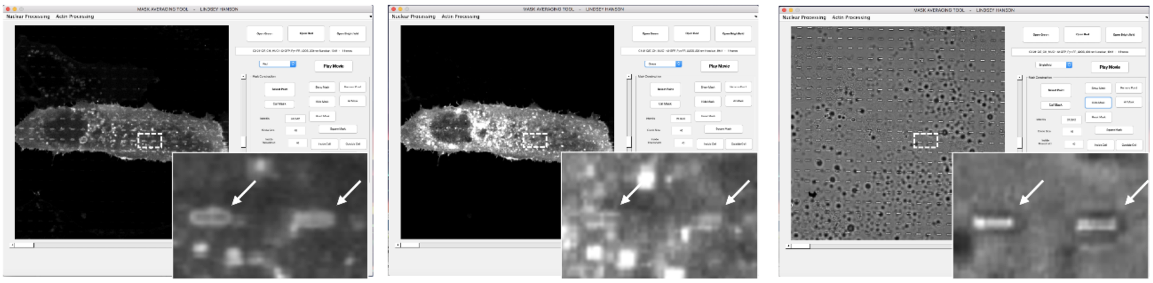

Step 2

mCherry-CAAX channel | MUC1-42TR-GFP channel | Bright Field channel

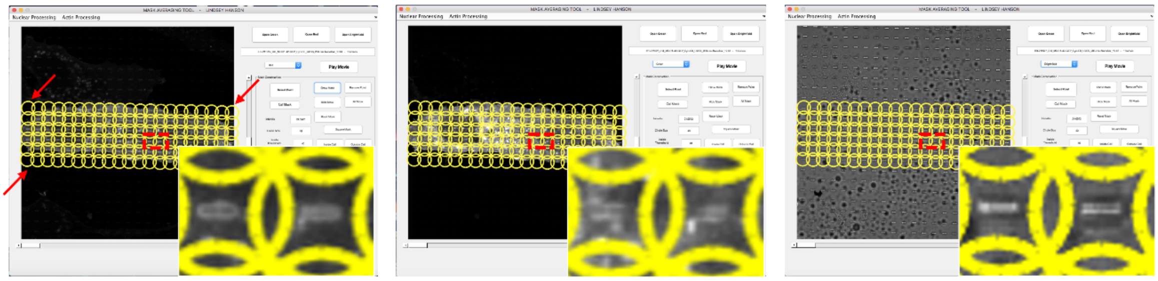

Step 3

mCherry-CAAX channel | MUC1-42TR-GFP channel | Bright Field channel

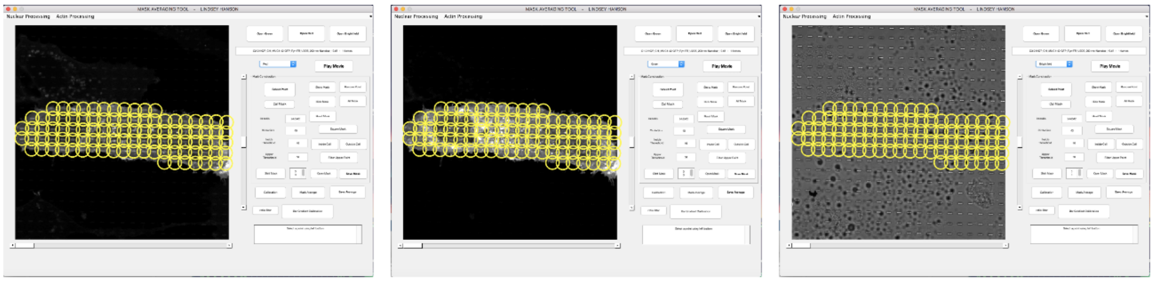

Step 4

mCherry-CAAX channel | MUC1-42TR-GFP channel | Bright Field channel

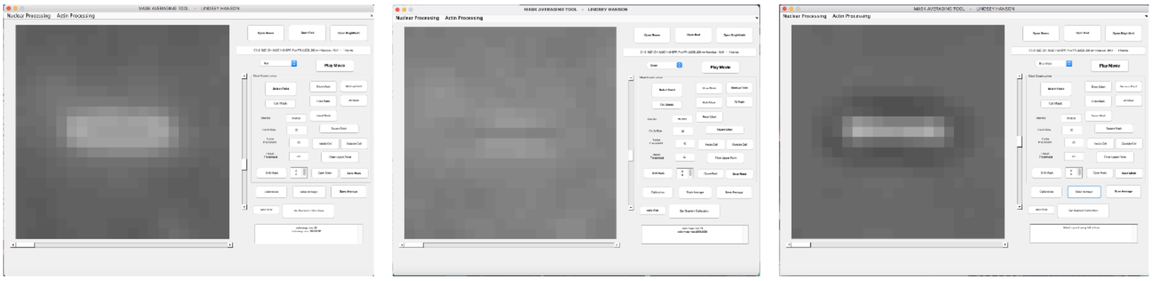

Step 5

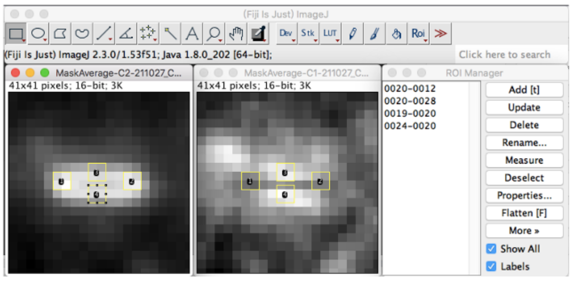

Step 6

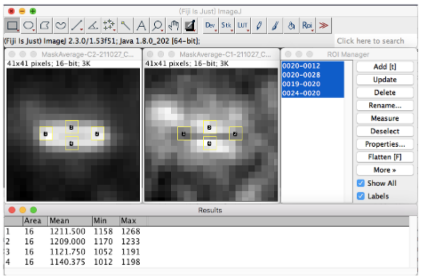

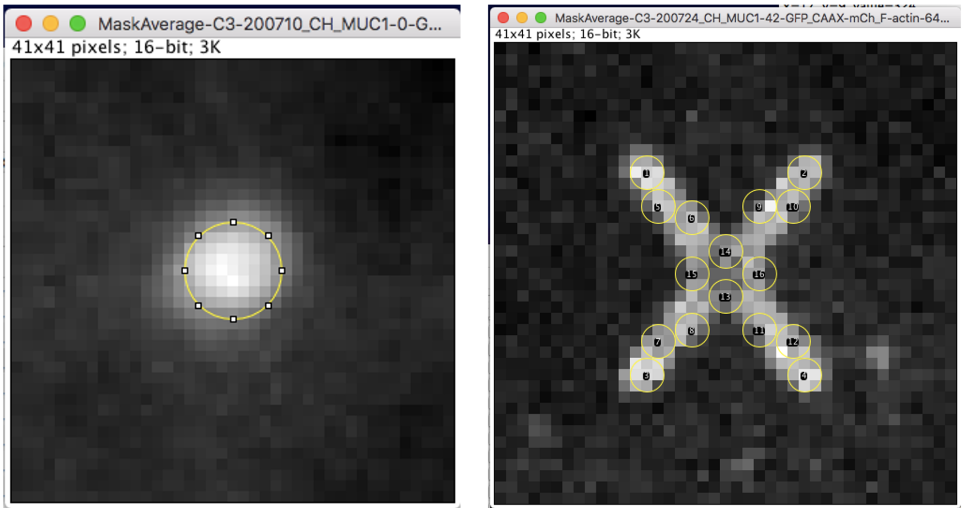

Supplementary Figure 2. A detailed description for the quantification of fluorescence signals of proteins on nanostructured substrates. The confocal fluorescence images were processed and analyzed using ImageJ and customized MATLAB programs. To quantify the curvature preference of a protein of interest, the intensity can be normalized by the membrane intensity at the same location (for nanopillars experiments), or nanostructures with internal references (such as the sidewalls of nanobars or nanoXs) can be used.

Procedure (use nanobar experiment as an example):

1. Load three-channel (GFP, mCherry, and bright field) images taken from the same field.
2. Manually click on the center of three nanobars (red arrows) in the mCherry-CAAX channel. Since the distance between nanobars is fixed, the software automatically locates all the nanobars in a rectangular array (yellow circles). Next, the software automatically propagates the nanopillar locations to all three color channels (GFP, mCherry, and bright field).
3. By intensity thresholding in the mCherry channel, the software removes nanobars that are located outside the cell of interest. In some cases, a nanobar outside the cell needs to be manually removed by clicking anywhere inside its yellow circle.
4. Based on the nanobar locations, the software automatically creates an averaged nanobar image from all the nanobars inside a cell. The cell in the example image interacts with 100 nanobars. In general, each cell contacts ~30-150 nanobars. An average nanobar image is created for each color channel for the selected cell.
5. Four ROIs, two located at the ends of the nanobar and two located at the side walls of the nanobar, are created on the membrane mCherry channel of the averaged nanobar image. The same ROI locations are re-created on the GFP channel. The same ROIs are used for all cells.
6. From the ROIs, the nanobar end-to-side ratios are independently calculated for mCherry and GFP channels. Then, the ratio for the MUC1_42TR-GFP channel (green) is divided by the ratio for the mCherry-CAAX channel (red). This step normalizes the protein ratio to the membrane ratio, which helps to distinguish whether the protein truly has a curvature preference vs. whether there is a higher protein signal due to more membranes at curved locations.

- Repeat the step 1-3 for nanobars of other sizes if necessary.
- For nanopillar experiments, create a small circular ROI to cover the fluorescence signal at nanopillars. The region surrounding the nanopillar ROI is used for assessing cytosolic background. Calculate the intensity ratio by dividing the fluorescence signal at nanopillars by that of the surrounding.
- For nanoX experiments, create 16 small ROIs to cover the centers of 4 ends, 8 side walls (2 for each arm) and 4 inner faces. Measure the fluorescence intensities in the 16 ROIs and compute the end-to-side, two inner-to-side intensity ratios.

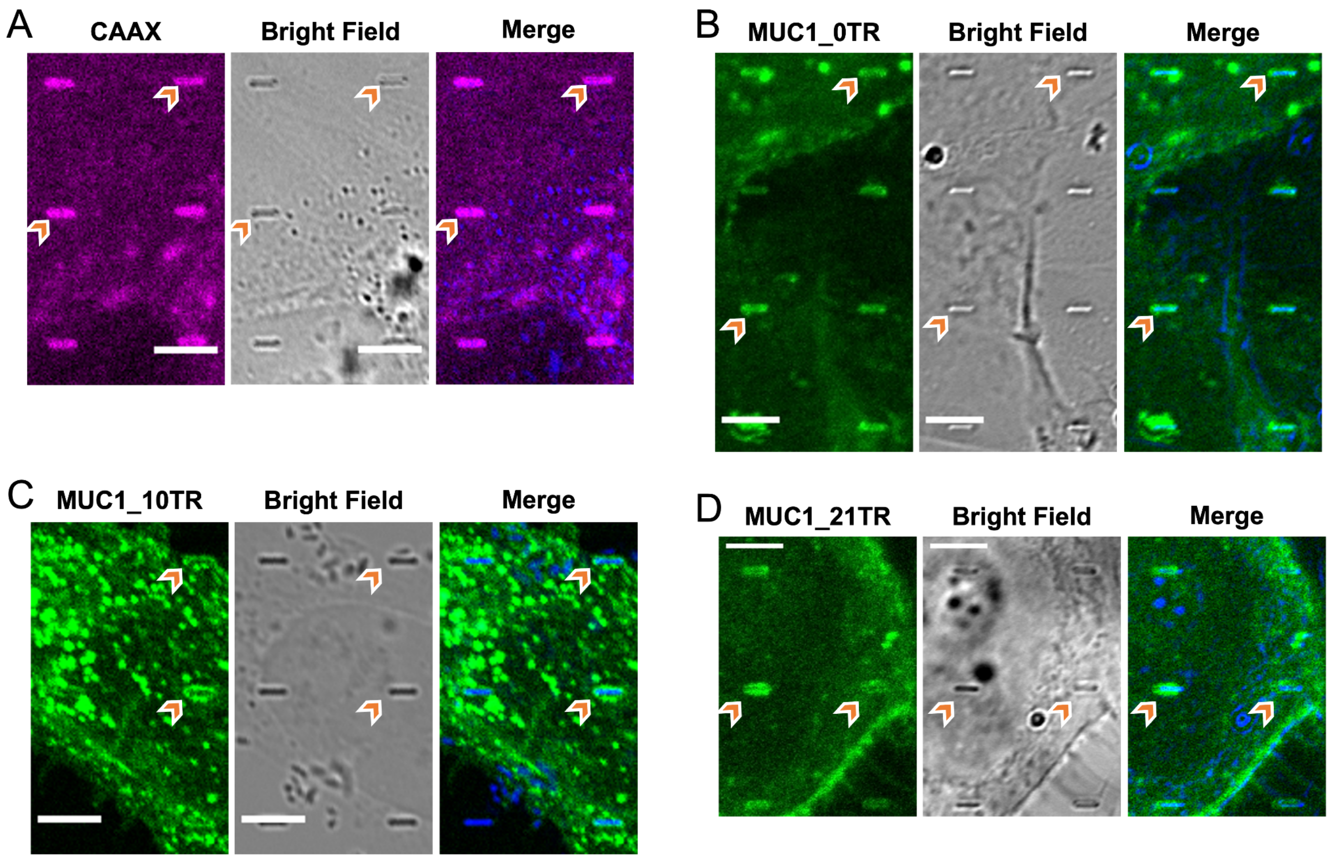

Supplementary Figure 3. MUC1-ΔCT-GFP-transfected U2OS cells on the 200-nm nanobar arrays.

Zoom-in confocal images of **(A)** mCherry-CAAX-transfected, **(B)** MUC1-ΔCT_0TR-GFP-transfected, **(C)** MUC1-ΔCT_10TR-GFP-transfected, and **(D)** MUC1-ΔCT_21TR-GFP-transfected U2OS cells cultured on the 200-nm nanobar arrays. Bright field images of the nanobar in the merge subsets were converted into blue color for visualization purposes. Scale bars represent 5 μm. Arrows were drawn for guidance purposes.

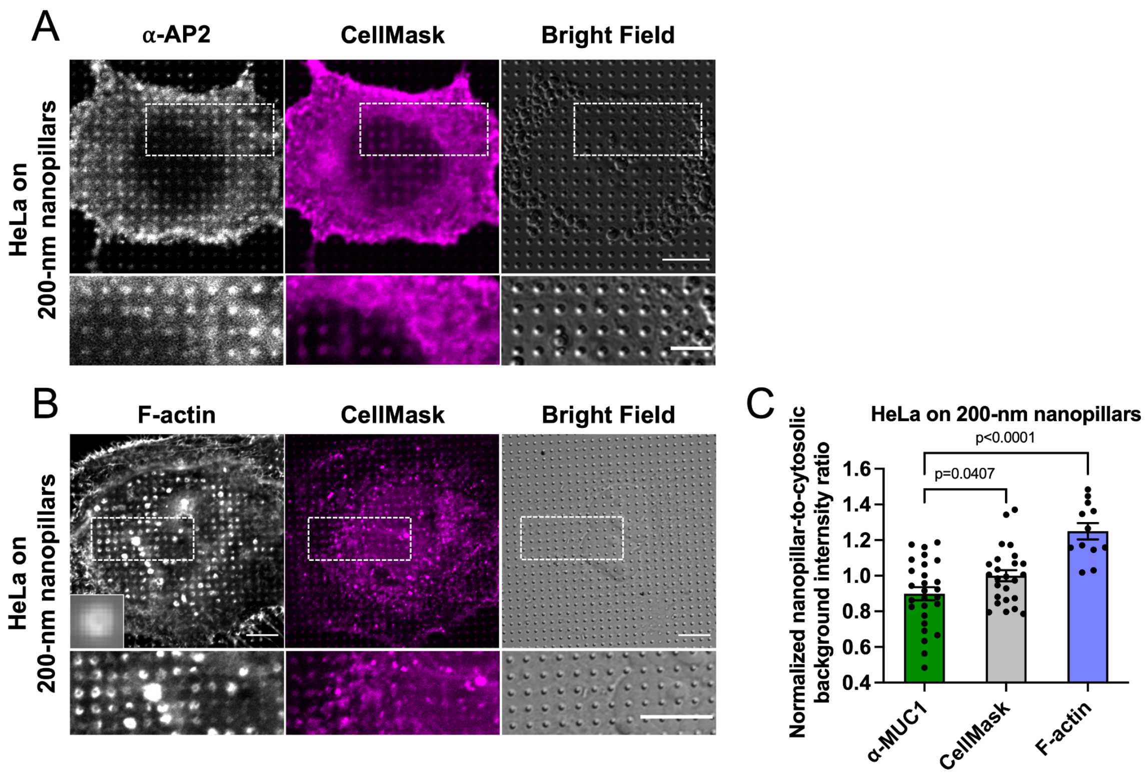

Supplementary Figure 4. AP2 and F-actin staining in U2OS or Hela cells on the 200-nm nanopillar arrays.

(A-B) Confocal images show accumulation of (A) α-AP2 and (B) F-actin on the 200-nm-diameter nanopillars in Hela cells. The square inset is the averaged images of proteins distributed on the nanopillars. **(C)** Quantification of α-MUC1, CellMask and F-actin signals on Hela cells plated on the 200-nm nanopillar arrays (see Supplementary Table 2A for the detailed statistics). The ratios for α-MUC1 and CellMask are from Fig. 1N. All ratios have been normalized against the CellMask signals. The spacing and height of the 200-nm nanopillar arrays are 2.5 µm and 1 µm, respectively. Scale bars represent 10 µm. F-actin was stained with phalloidin. Welch's t tests (unpaired, two-tailed, not assuming equal variance) are applied for all statistical analyses in this figure. Error bars represent SEM.

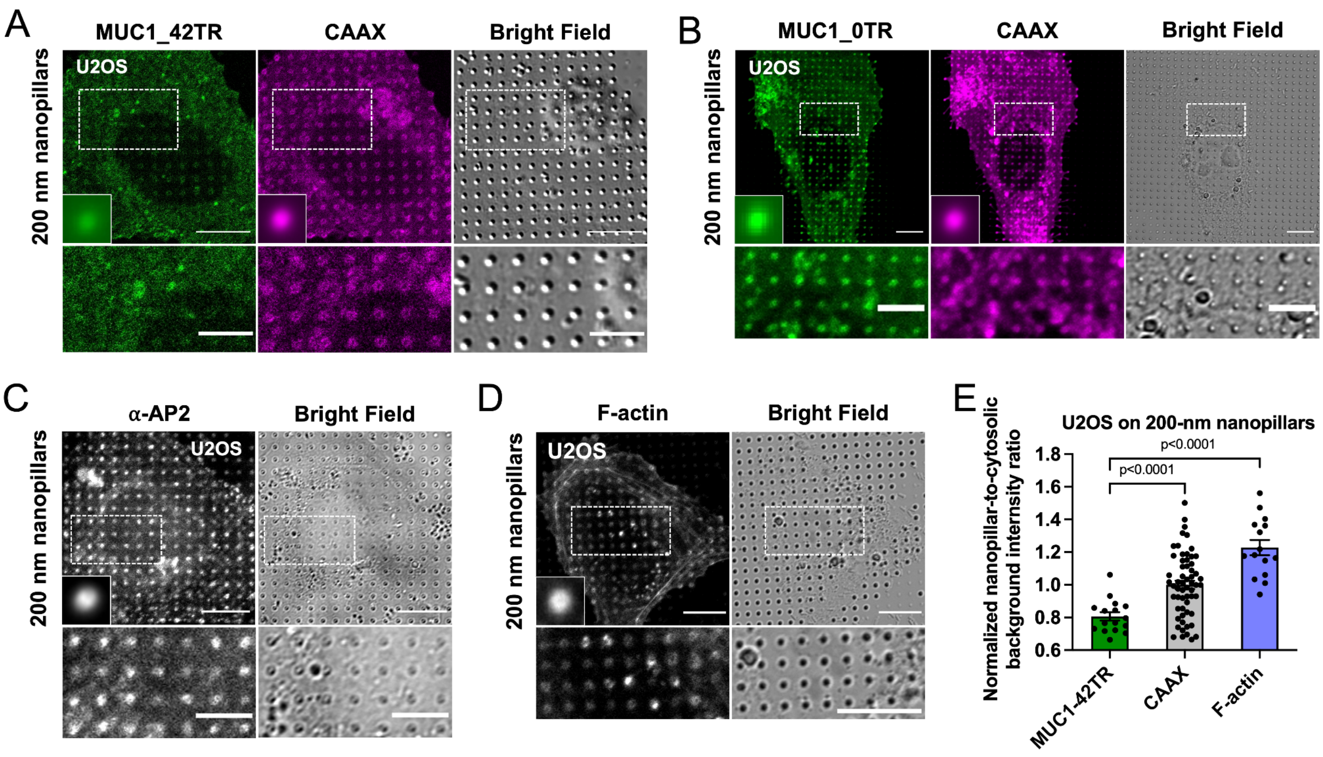

Supplementary Figure 5. MUC1-ΔCT-GFP-transfected U2OS cells on the 200-nm nanopillar arrays.

Confocal images of **(A)** MUC1-ΔCT_42TR-GFP- and mCherry-CAAX-cotransfected U2OS cells, **(B)** MUC1-ΔCT_0TR-GFP- and mCherry-CAAX-cotransfected U2OS cells, **(C)** U2OS cells stained with anti-AP2 antibodies and **(D)** U2OS cells stained with phalloidin cultured on the 200-nm nanopillar arrays. The square insets are the averaged images of proteins distributed on the nanopillars. **(E)** Quantification of MUC1-ΔCT_42TR-GFP, CellMask and F-actin signals in U2OS cells plated on the 200-nm nanopillar arrays (see Supplementary Table 2B for the detailed statistics). The ratios for α-MUC1 and CAAX are from Fig. 1O. All ratios have been normalized against the mCherry-CAAX signals. The spacing and height of the 200-nm nanopillar arrays are 2.5 μm and 1 μm, respectively. Scale bars represent 10 μm; Scale bars in the zoom-in images represent 5 μm. Welch's t tests (unpaired, two-tailed, not assuming equal variance) are applied for all statistical analyses in this figure.

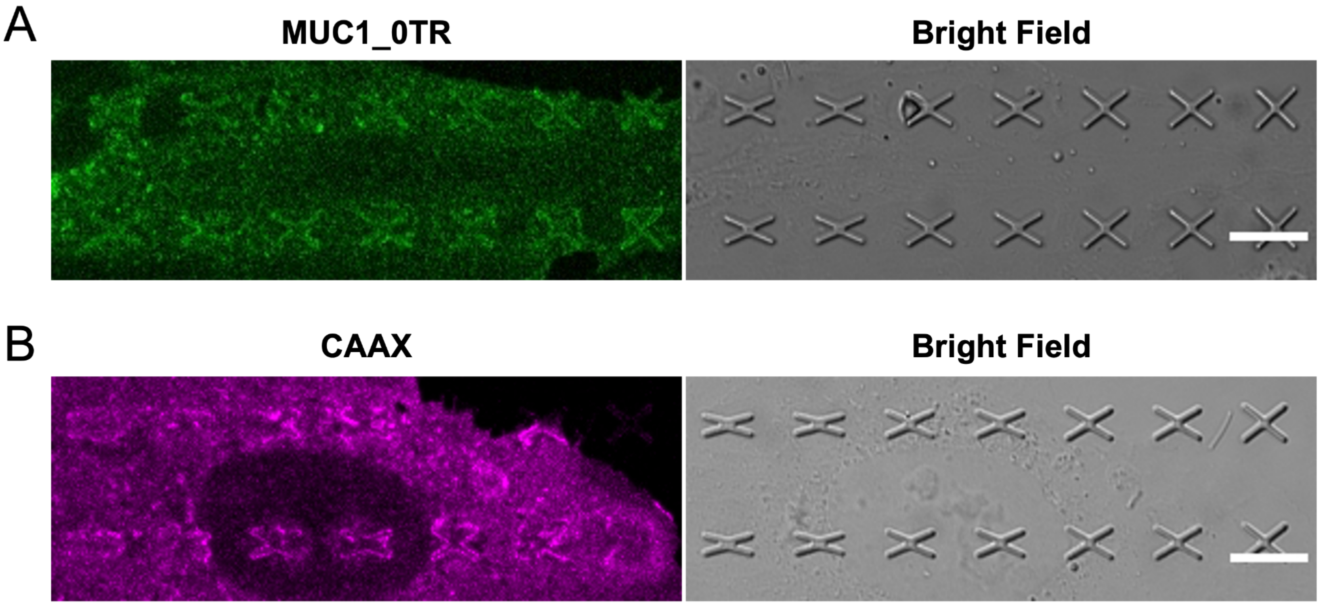

Supplementary Figure 6. MUC1-ΔCT_0TR-GFP-transfected U2OS cells on the gradient nanoX arrays.

Confocal images of **(A)** MUC1-ΔCT_0TR-GFP-transfected U2OS cells and **(B)** mCherry-CAAX-transfected U2OS cells cultured on the gradient nanoX arrays. All nanoX are 350 nm in width, 2 μm in height and 10 μm in spacing. NanoX inner angle (θ) increment: 15º. Scale bars represent 10 μm.

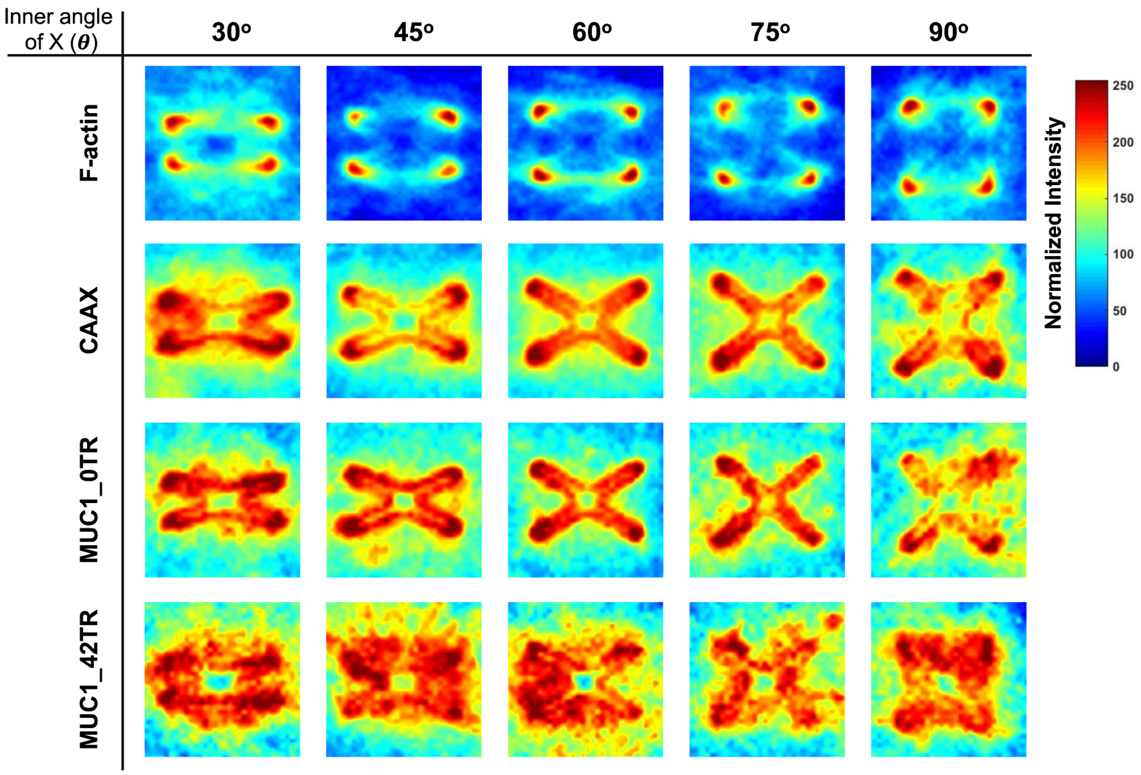

Supplementary Figure 7. Heatmaps depicting the intensity distribution of mCherry-CAAX, two MUC1-Δ CT-GFP, and F-actin signals in U2OS cells plated on the gradient nanoX arrays (Cell-based experiments).

All nanoX are 350 nm in width, 2 µm in height and 10 µm in spacing. NanoX inner angle (θ) increment: 15°.

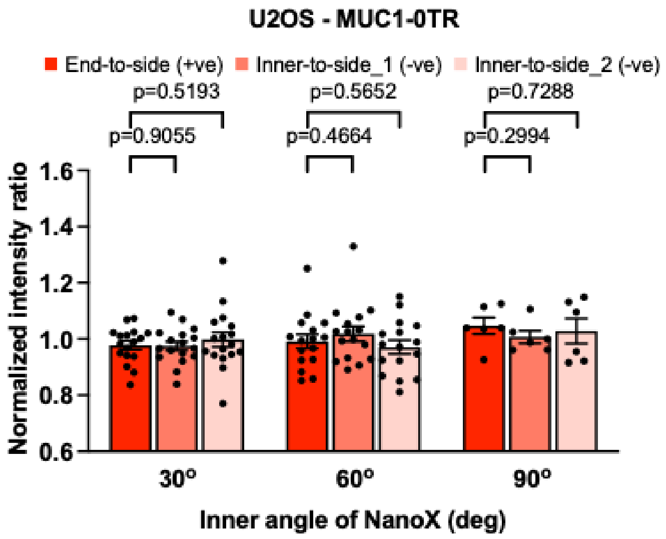

Supplementary Figure 8. Quantification of MUC1-ΔCT-0TR-GFP signals in U2OS cells plated on the gradient nanoX arrays. (Cell-based experiments).

All ratios have been normalized against the mCherry-CAAX signals (see Supplementary Table 3B for the detailed statistics). Welch's t tests (unpaired, two-tailed, not assuming equal variance) are applied for all statistical analyses in this figure. Error bars represent SEM.

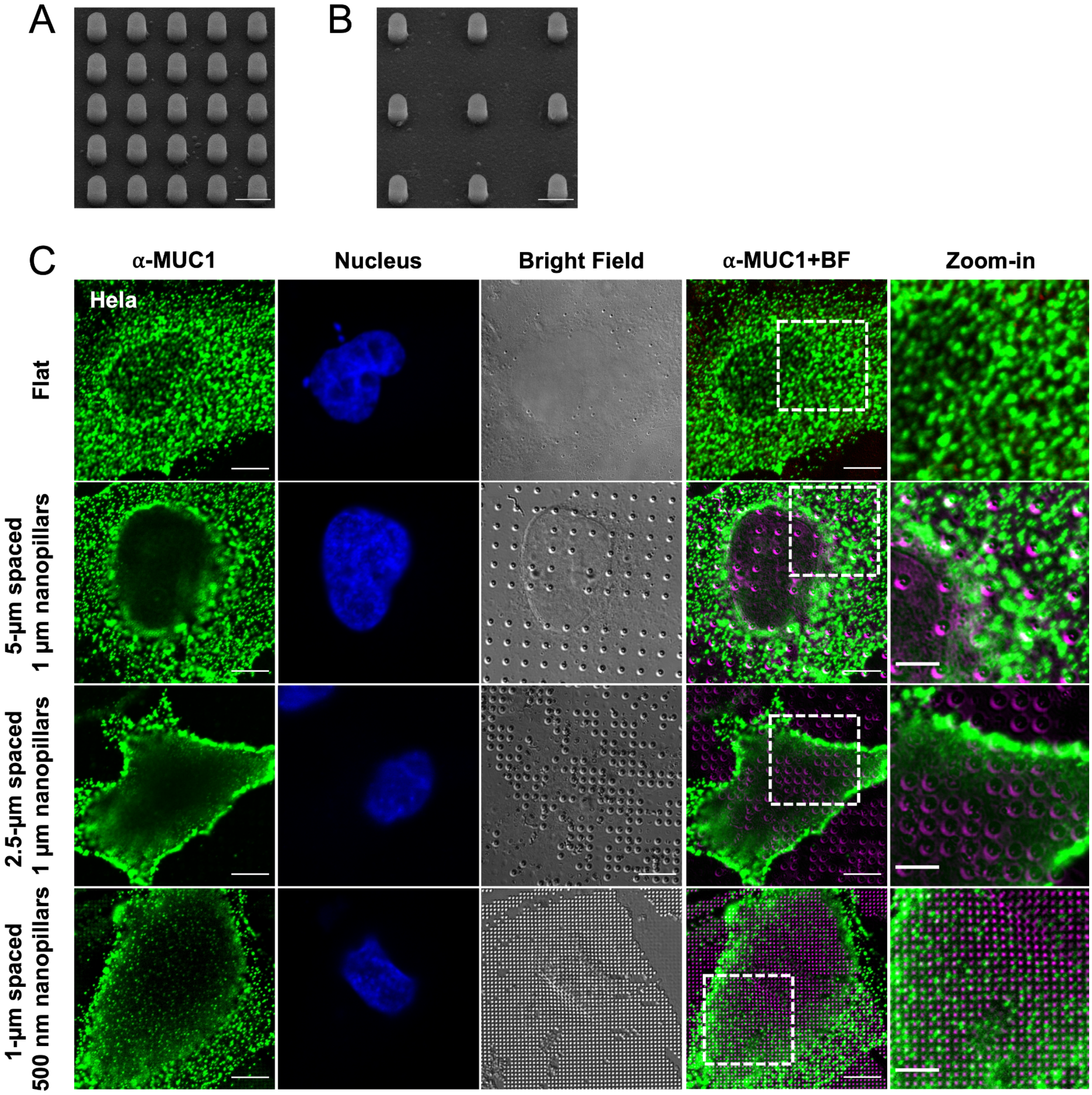

Supplementary Figure 9. Confocal images of Hela cells plated on the nanopillar substrates of various spacings.

(A) A SEM image of the 1-μm-diameter, 1-μm-height, 2.5-μm-spaced nanopillar arrays. The images were taken with a stage tilt of 45°. Scale bar represents 2 μm. **(B)** A SEM image of the 1-μm-diameter, 1-μm-height, 5-μm-spaced nanopillar arrays. The images were taken with a stage tilt of 45°. Scale bar represents 2 μm. **(C)** Confocal images of Hela cells plated on the nanopillar arrays of various spacings. MUC1 was immunostained with mouse anti-MUC1/episialin antibody (214D4) and fluorescently-labeled goat anti-mouse antibody, sequentially; Nuclei were visualized via Hoechst stain. Scale bars=10 μm for the whole-cell images; 5 μm for the zoom-in images. The bright field channel in the merge image is background-subtracted and converted into magenta color for visualization purposes.

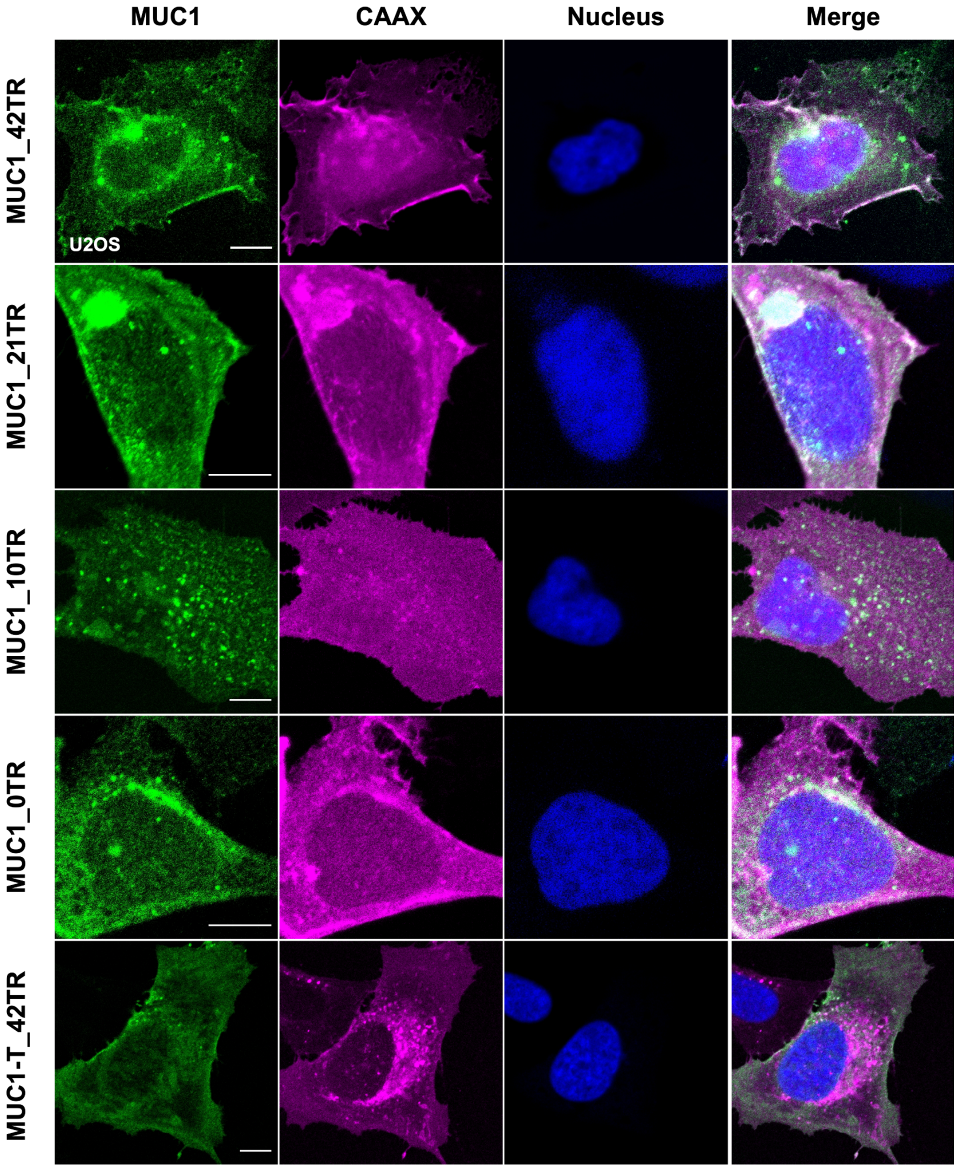

Supplementary Figure 10. Co-transfection of MUC1-ΔCT-GFP of varying lengths and mCherry-CAAX in U2OS cells.

Cells were all cultured on flat surfaces. Nuclei were visualized via Hoechst stain. Scale bars represent 10 μm.

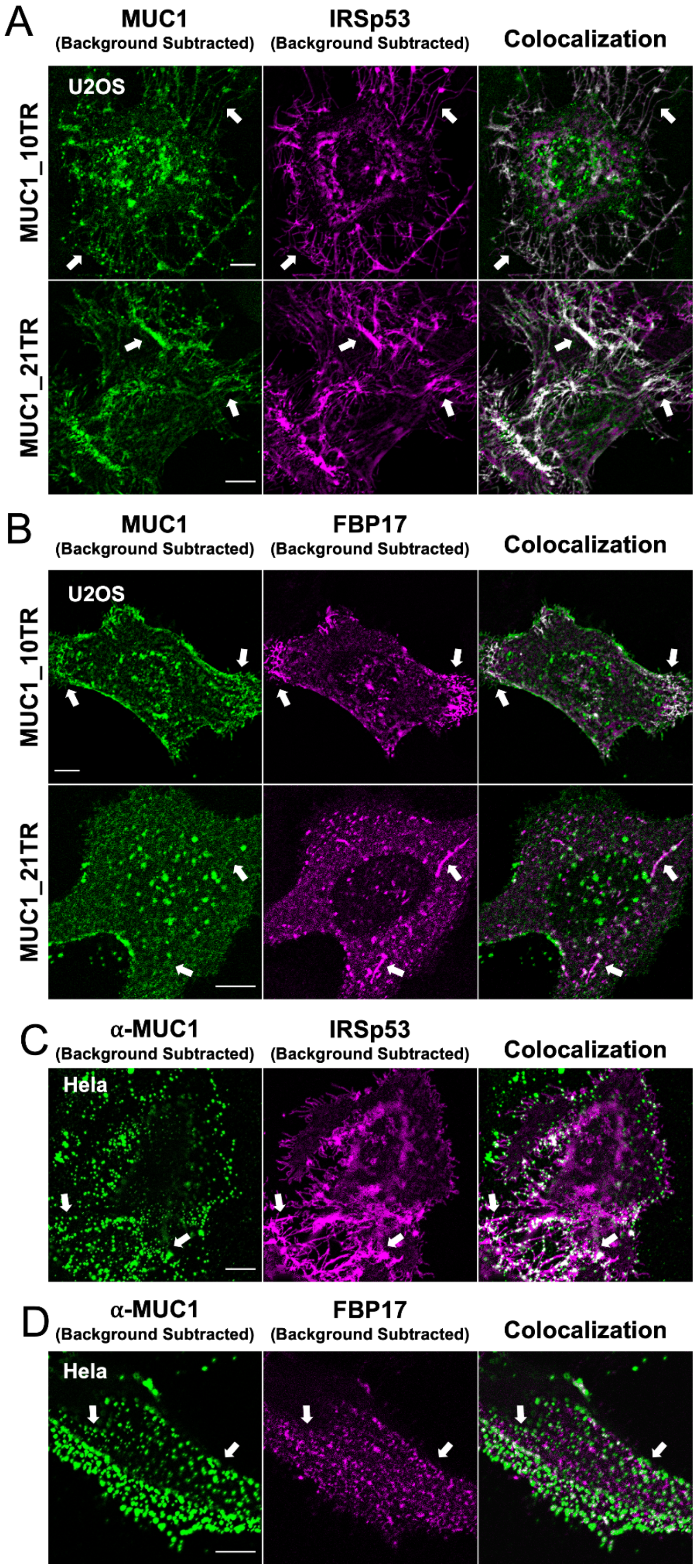
A
MUC1
(Background Subtracted)
IRSp53
(Background Subtracted)
Colocalization
U2OS
MUC1_10TR
MUC1_21TR
B
MUC1
(Background Subtracted)
FBP17
(Background Subtracted)
Colocalization
U2OS
MUC1_10TR
MUC1_21TR
C
α-MUC1
(Background Subtracted)
IRSp53
(Background Subtracted)
Colocalization
Hela
D
α-MUC1
(Background Subtracted)
FBP17
(Background Subtracted)
Colocalization
Hela

Supplementary Figure 11. MUC1 avoids positively-curved membranes induced by membrane-sculpturing proteins.

(A-B) Confocal images of U2OS cells transfected with either MUC1-ΔCT_10TR-GFP or MUC1-ΔCT_21TR-GFP and co-transfected with (A) IRSp53-mCherry to induce membrane protrusions with negative curvature; or (B) mCherry-FBP17-ΔSH3 to generate membrane invaginations with positive curvature. Scale bars represent 10 µm. **(C-D)** MUC1 immunostaining on the (C) IRSp53-mCherry- (D) mCherry-FBP17-ΔSH3-transfected Hela cells. Scale bars represent 10 µm. In (C) and (D), MUC1 was immunostained with mouse anti-MUC1/episialin antibody (214D4) and fluorescently-labeled goat anti-mouse antibody, sequentially. All cells were cultured on flat surfaces. Arrows were drawn for guidance purposes.

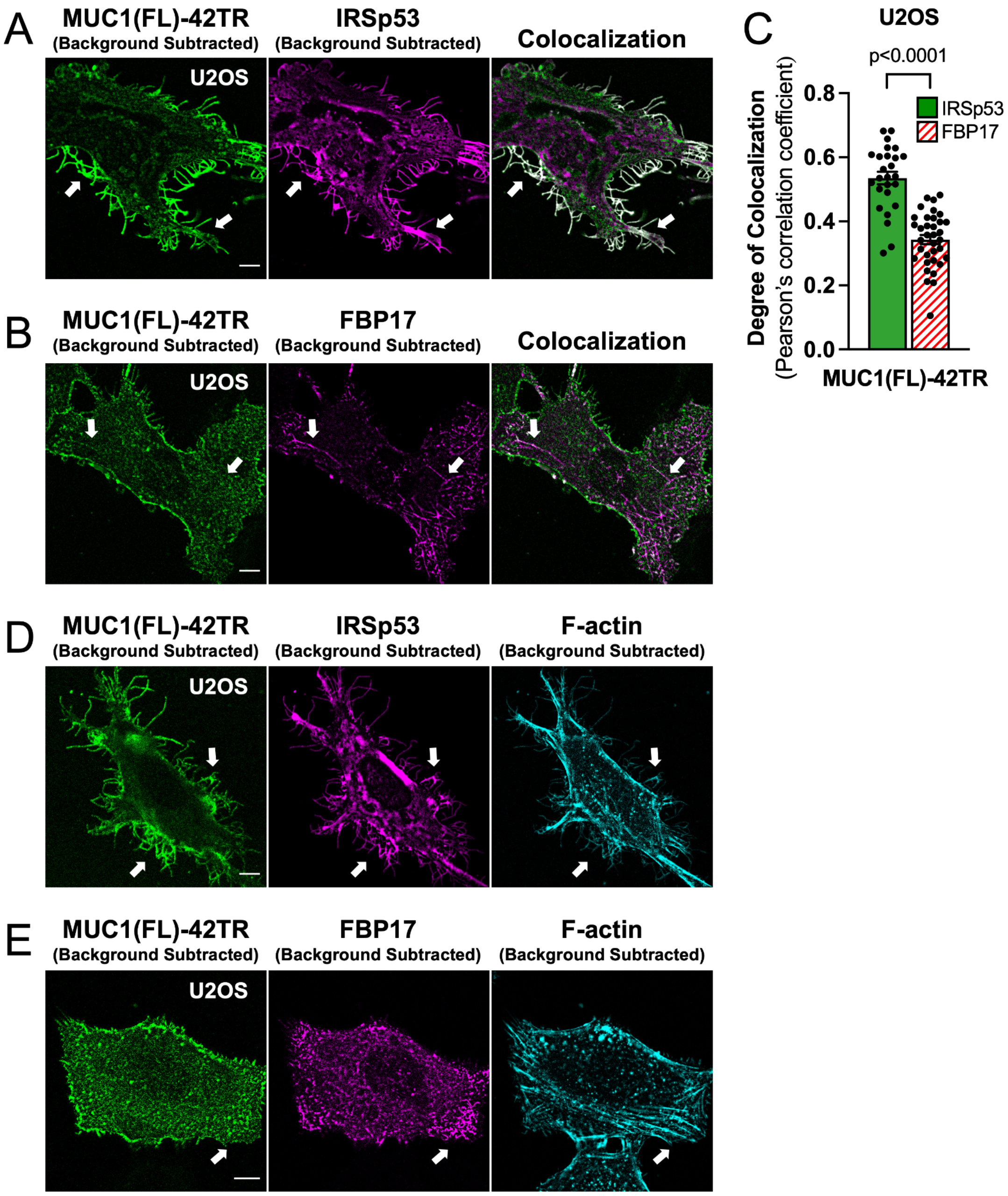

Supplementary Figure 12. Full-length MUC1 also avoids positively-curved membranes and prefers negatively-curved ones induced by membrane-sculpturing proteins.

(A-B) Confocal images of U2OS cells transfected with MUC1(FL)_42TR-GFP and co-transfected with (A) IRSp53-mCherry to induce membrane protrusions with negative curvature; or (B) mCherry-FBP17-ΔSH3 to generate membrane invaginations with positive curvature. Scale bars represent 10 μm. **(C)** Colocalization analysis of MUC1(FL)_42TR-GFP and two mCherry-BAR-family proteins in U2OS cells (see Supplementary Table 4D for the detailed statistics). Arrows were drawn for guidance purposes. **(D-E)** Confocal images of U2OS cells co-transfected with (D) MUC1(FL)_42TR-GFP and IRSp53-mCherry or (E) MUC1(FL)_42TR-GFP and mCherry-FBP17-ΔSH3 and stained with phalloidin to visualize F-actin. Scale bars represent 10 μm. All cells were cultured on flat surfaces. Welch's t tests (unpaired, two-tailed, not assuming equal variance) are applied for all statistical analyses in this figure. Error bars represent SEM. Arrows were drawn for guidance purposes.

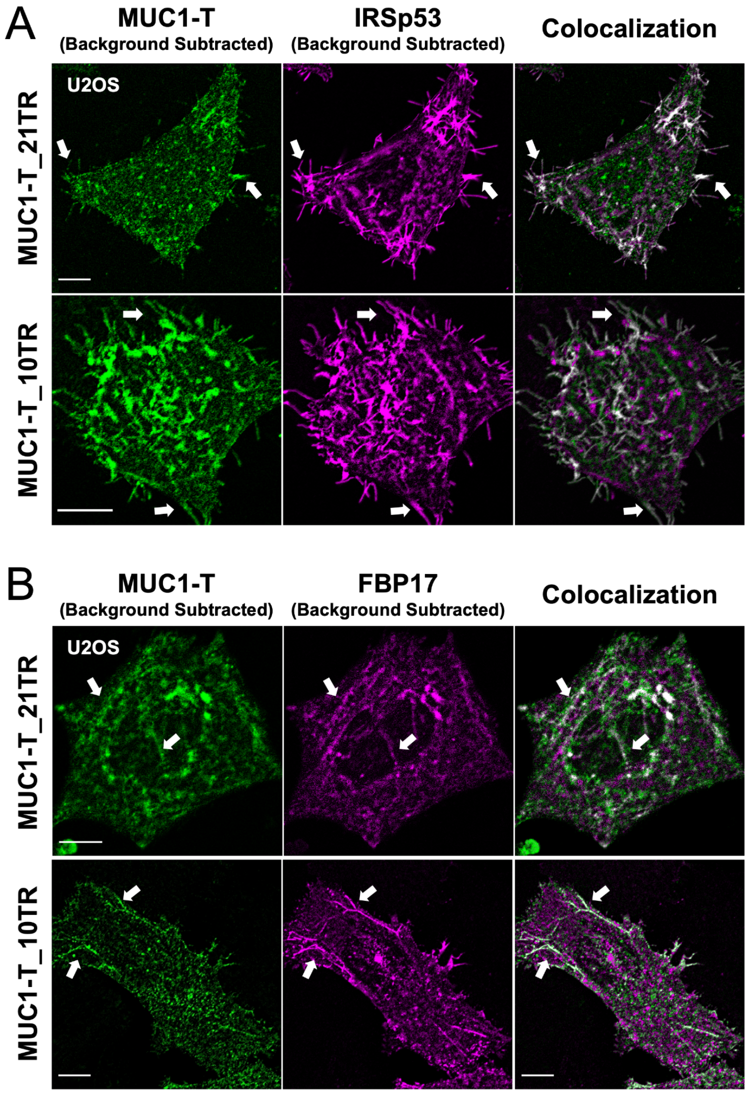

Supplementary Figure 13. Reduced glycosylation of MUC1 causes reduced sensitivity toward curvatures induced by membrane-sculpturing proteins.

(A) Confocal images of U2OS cells co-transfected with IRSp53-mCherry and either MUC1-ΔCT-T_10TR-GFP or MUC1-ΔCT-T_21TR-GFP. Scale bars represent 10 μm. **(B)** Confocal images of U2OS cells co-transfected with mCherry-FBP17-ΔSH3 and either either MUC1-ΔCT-T_10TR-GFP or MUC1-ΔCT-T_21TR-GFP. Scale bars represent 10 μm. All cells were cultured on the flat surface. Arrows were drawn for guidance purposes.

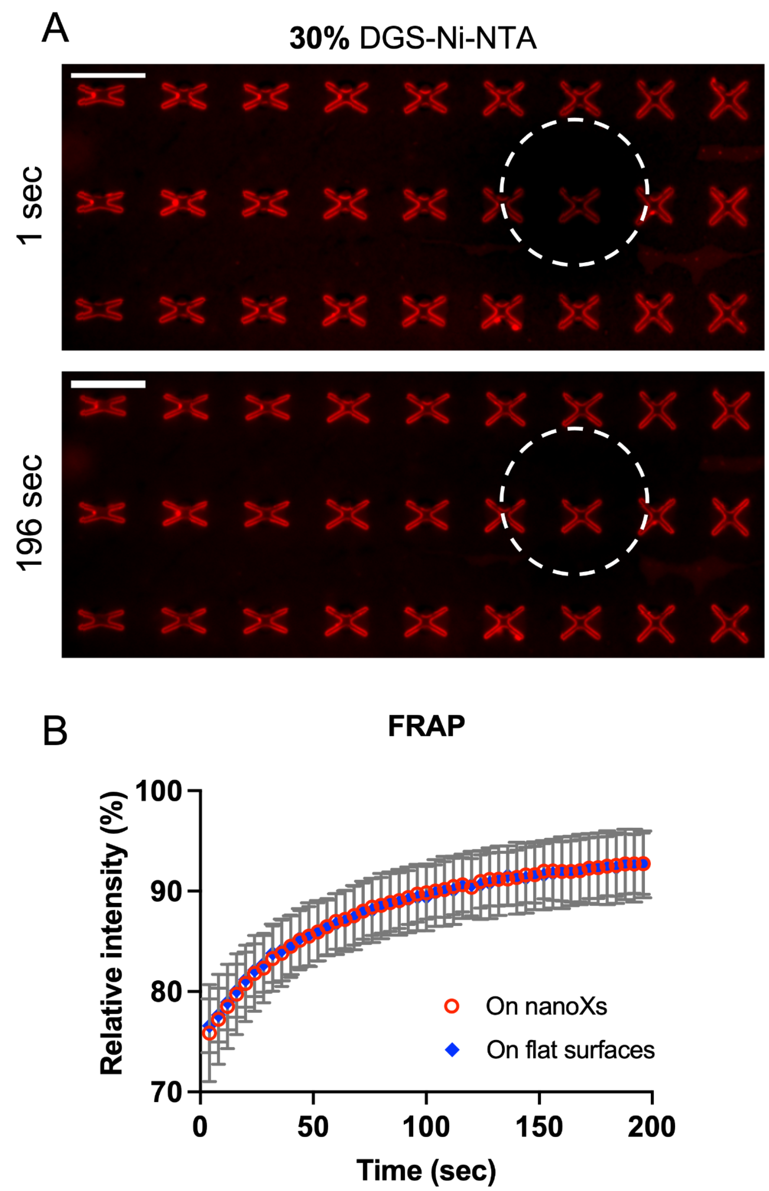

Supplementary Figure 14. Lipid bilayer fluidity on the gradient nanoX arrays was measured by Fluorescence Recovery After Photobleaching (FRAP) assay (SLB experiments).

(A) Fluorescence images of the lipid bilayers on the gradient nanoX arrays at 1 sec and 196 sec after photobleaching. The bilayers were doped with 30% DGS-Ni-NTA and ~1 mol.% of Texas Red-tagged DHPE for visualization. White-dashed circles indicate the bleached regions. **(B)** A plot of the time trace of fluorescence recovery signals shows that the lipid fluidity on nanoXs is comparable to that on flat surfaces. Each data point is averaged from 5 fields of view. Error bars represent SD.

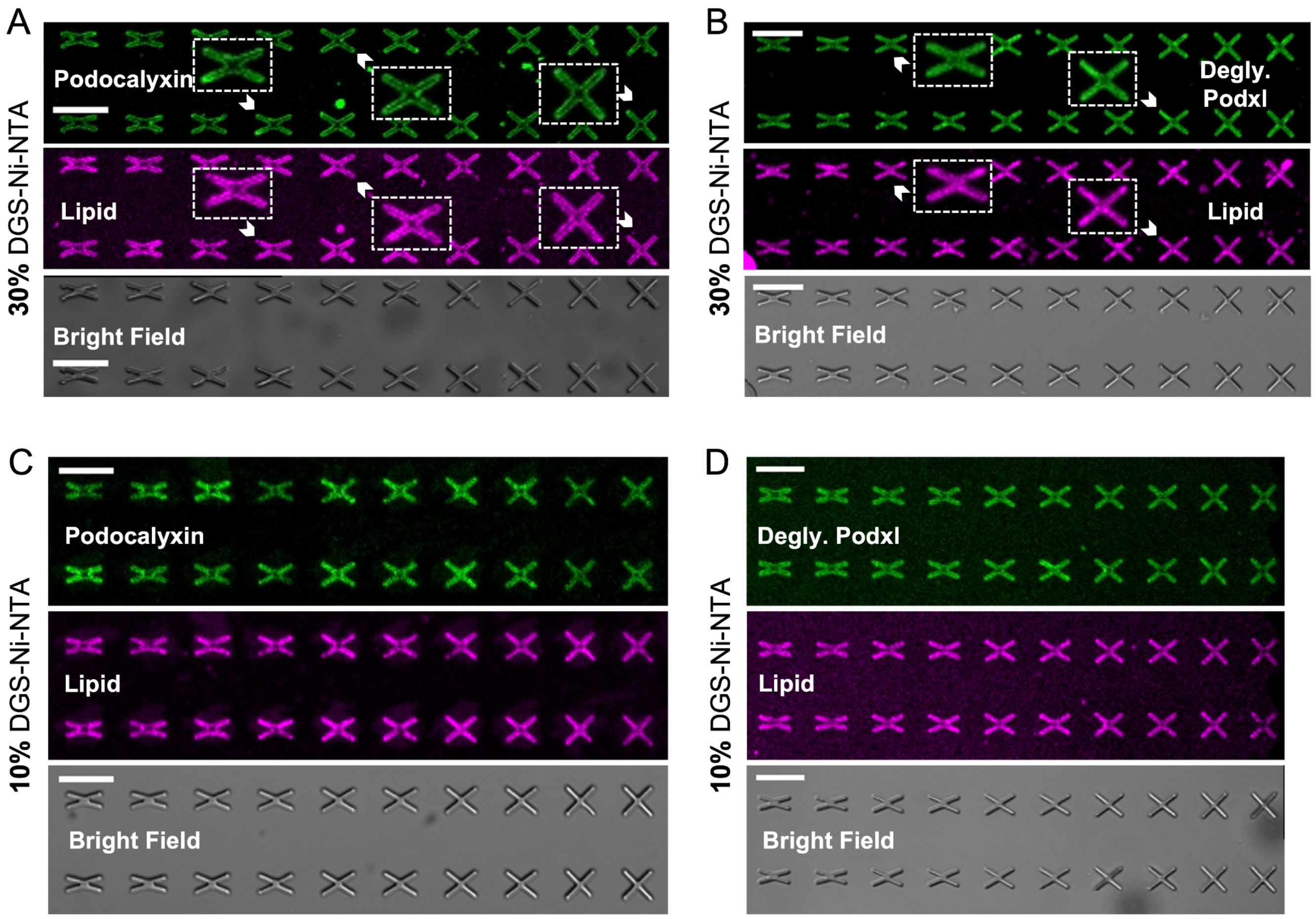

Supplementary Figure 15. Confocal images of fluorescently-labeled Podocalyxin and deglycosylated Podocalyxin on the SLB-coated gradient nanoX arrays doped with 10% DGS-Ni-NTA.

(A-B) Confocal images of fluorescently-labeled (A) Podocalyxin (Podxl) and (B) deglycosylated Podocalyxin on the SLB-coated gradient nanoX arrays. The lipid mixture was doped with 30% DGS-Ni-NTA and ~1 mol.% of Texas Red-tagged DHPE as a lipid bilayer marker. The inner angle of nanoX ranges from 30° (left) to 90° (right). All nanoX are 350 nm in width, 2 μm in height and 10 μm in spacing. NanoX inner angle (θ) increment: 15°. Scale bars represent 10 μm. **(C-D)** Confocal images of fluorescently-labeled (C) Podxl and (D) deglycosylated Podxl on the SLB-coated gradient nanoX arrays. The lipid mixture was doped with 10% DGS-Ni-NTA and ~1 mol.% of Texas Red-tagged DHPE as a lipid bilayer marker. Scale bars represent 10 μm.

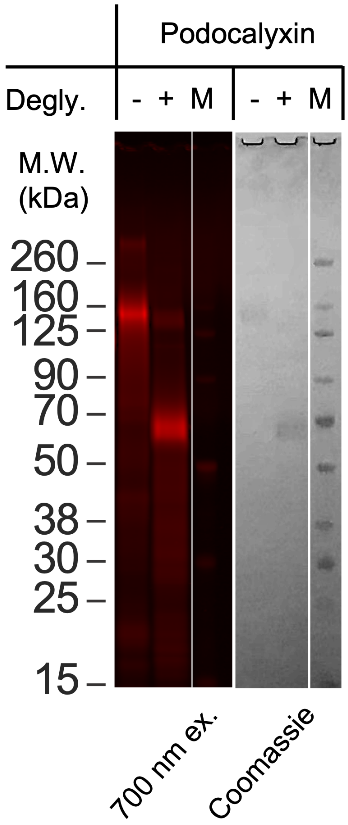

Supplementary Figure 16. Polyacrylamide gel images of native and deglycosylated recombinant Podocalyxin protein with a His-tag.

The protein was labeled with Alexa Fluor 647. The imaging was resolved at the excitation wavelength of 700 nm. The uncut gels are provided in the Source Data. (Abbreviations: ‘M.W. for molecular weight; ‘M’ for markers)

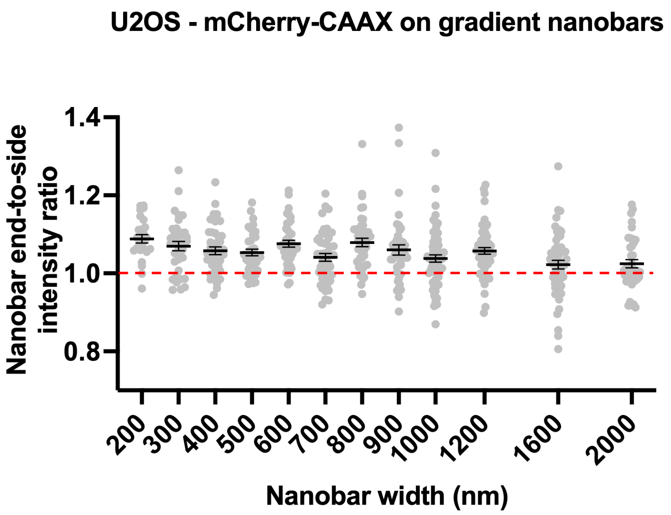

Supplementary Figure 17. Quantification of mCherry-CAAX signals in U2OS cells cultured on the gradient nanobar arrays. Error bars represent SEM.

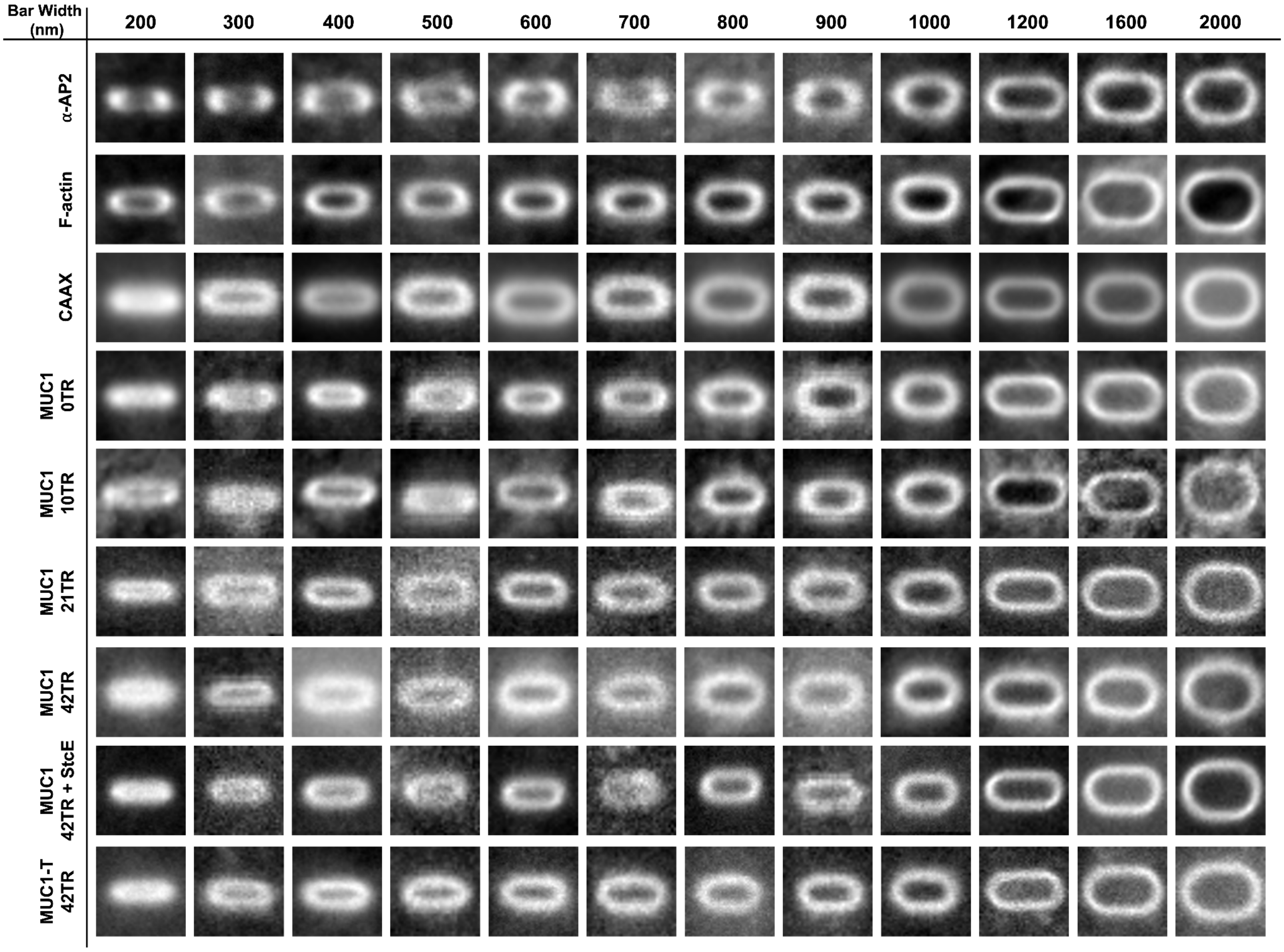

Supplementary Figure 18. Averaged fluorescence images of α-AP2, F-actin, mCherry-CAAX and 6 different MUC1-ΔCT-GFP signals in U2OS cells plated on the gradient nanobar arrays.

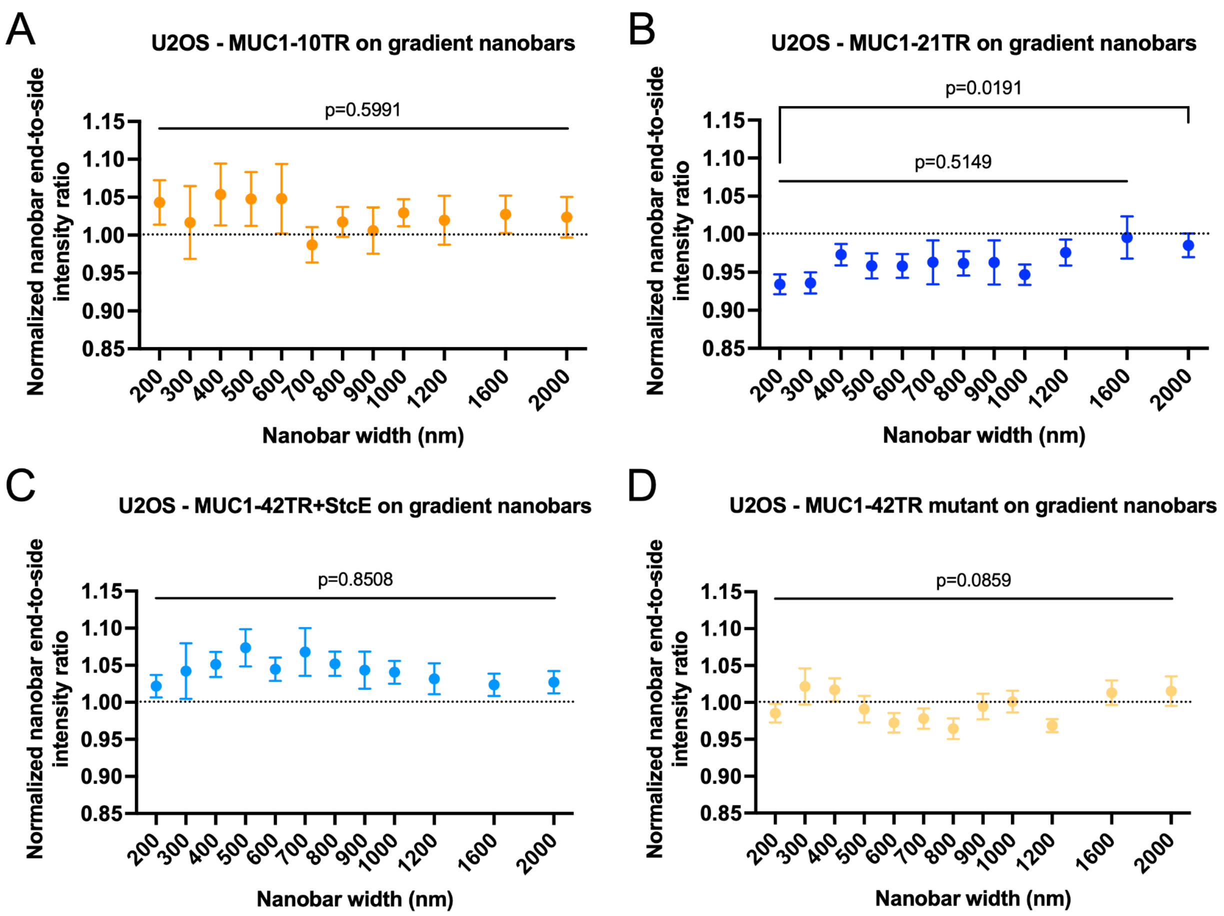

Supplementary Figure 19. Quantification of MUC1-ΔCT-GFP signals in U2OS cells cultured on the gradient nanobar arrays.

Quantification of **(A)** MUC1-ΔCT_10TR-GFP, **(B)** MUC1-ΔCT_21TR-GFP, **(C)** StcE-treated MUC1-ΔCT_42TR-GFP and **(D)** MUC1-ΔCT_42TR-GFP triple mutants on the gradient nanobar arrays. All ratios have been normalized against mCherry-CAAX signals (see Supplementary Table 7 for the detailed statistics). Both Welch's t tests (unpaired, two-tailed, not assuming equal variance) and one-way Welch's ANOVA are applied for the statistical analyses in this figure. Error bars represent SEM.
