# Supplementary material for: Membrane curvature regulates the spatial distribution of bulky glycoproteins
Source: Nat Commun. 2022 Jun 2;13:3093. doi: 10.1038/s41467-022-30610-2 (PMC9163104; doi:10.1038/s41467-022-30610-2)
Supplement: Supplementary file 3 — Reporting Summary [file 41467_2022_30610_MOESM3_ESM.pdf]

Corresponding author(s): Cui, Bianxiao

Last updated by author(s): Apr 14, 2022

Reporting Summary

Nature Portfolio wishes to improve the reproducibility of the work that we publish. This form provides structure for consistency and transparency in reporting. For further information on Nature Portfolio policies, see our Editorial Policies and the Editorial Policy Checklist.

Statistics

For all statistical analyses, confirm that the following items are present in the figure legend, table legend, main text, or Methods section.

n/a	Confirmed	
☐	☒	The exact sample size (*n*) for each experimental group/condition, given as a discrete number and unit of measurement
☐	☒	A statement on whether measurements were taken from distinct samples or whether the same sample was measured repeatedly
☐	☒	The statistical test(s) used AND whether they are one- or two-sided<br>*Only common tests should be described solely by name; describe more complex techniques in the Methods section.*
☒	☐	A description of all covariates tested
☐	☒	A description of any assumptions or corrections, such as tests of normality and adjustment for multiple comparisons
☐	☒	A full description of the statistical parameters including central tendency (e.g. means) or other basic estimates (e.g. regression coefficient) AND variation (e.g. standard deviation) or associated estimates of uncertainty (e.g. confidence intervals)
☐	☒	For null hypothesis testing, the test statistic (e.g. *F*, *t*, *r*) with confidence intervals, effect sizes, degrees of freedom and *P* value noted<br>*Give P values as exact values whenever suitable.*
☒	☐	For Bayesian analysis, information on the choice of priors and Markov chain Monte Carlo settings
☒	☐	For hierarchical and complex designs, identification of the appropriate level for tests and full reporting of outcomes
☐	☒	Estimates of effect sizes (e.g. Cohen's *d*, Pearson's *r*), indicating how they were calculated

Our web collection on statistics for biologists contains articles on many of the points above.

Software and code

Policy information about availability of computer code

Data collection	MATLAB (Version R2018a, MathWorks)<br>Fiji (ImageJ2, version 2.3.0)
Data analysis	MATLAB (Version R2018a, MathWorks)<br>Fiji (ImageJ2, version 2.3.0)<br>Prism (Version 9, GraphPad software)<br>FCS Express™ 7 (De Novo Software)

For manuscripts utilizing custom algorithms or software that are central to the research but not yet described in published literature, software must be made available to editors and reviewers. We strongly encourage code deposition in a community repository (e.g. GitHub). See the Nature Portfolio guidelines for submitting code & software for further information.

Data

Policy information about availability of data

All manuscripts must include a data availability statement. This statement should provide the following information, where applicable:

- Accession codes, unique identifiers, or web links for publicly available datasets
- A description of any restrictions on data availability
- For clinical datasets or third party data, please ensure that the statement adheres to our policy

All data that support this work are included in the Source Data.

Field-specific reporting

Please select the one below that is the best fit for your research. If you are not sure, read the appropriate sections before making your selection.

☒ Life sciences ☐ Behavioural & social sciences ☐ Ecological, evolutionary & environmental sciences

For a reference copy of the document with all sections, see nature.com/documents/nr-reporting-summary-flat.pdf

Life sciences study design

All studies must disclose on these points even when the disclosure is negative.

Sample size	We didn't perform calculations to determine the sample sizes. We also listed the number of cells/nanostructures/fields of images considered for the analyses in the supplementary tables. Sample sizes were chosen based on the robustness of the results. The results were reproducible among independent experiments.
Data exclusions	No data was excluded.
Replication	All cell-based and in vitro experiments were confirmed with multiple replicates: All nanostructure-involved experiments were repeated at least three times; BAR family protein-involved experiments and endocytosis experiments were repeated at least twice. all attempts at replication were successful and reproducible.
Randomization	No other randomization was applied in this study except for the order of analysis for flow cytometric experiments.
Blinding	No blinding was performed in this work.

Reporting for specific materials, systems and methods

We require information from authors about some types of materials, experimental systems and methods used in many studies. Here, indicate whether each material, system or method listed is relevant to your study. If you are not sure if a list item applies to your research, read the appropriate section before selecting a response.

Materials & experimental systems

n/a	Involved in the study	
☐	☒	Antibodies
☐	☒	Eukaryotic cell lines
☒	☐	Palaeontology and archaeology
☒	☐	Animals and other organisms
☒	☐	Human research participants
☒	☐	Clinical data
☒	☐	Dual use research of concern

Methods

n/a	Involved in the study	
☒	☐	ChIP-seq
☐	☒	Flow cytometry
☒	☐	MRI-based neuroimaging

Antibodies

Antibodies used	mouse Anti-alpha Adaptin antibody [AP6] (anti-AP2, Abcam, #ab2730), 1:500<br>mouse anti-MUC1/episialin antibody (clone 214D4, Sigma-Aldrich, #05-652), 1:500<br>rabbit anti-GFP polyclonal antibody (Sigma-Aldrich, #PC408), 1:500<br>goat anti-mouse IgG Alexa Fluor 488 (Invitrogen, #A-11029), 1:1000<br>goat anti-mouse IgG Alexa Fluor 594 (Invitrogen, #A-11032), 1:1000<br>goat anti-mouse IgG Alexa Fluor 647 (Invitrogen, #A-21236), 1:1000<br>goat anti-rabbit IgG Alexa Fluor 647 (Invitrogen, #A-32733), 1:1000
Validation	mouse Anti-alpha Adaptin antibody [AP6] (anti-AP2, Abcam, #ab2730): https://www.abcam.com/alpha-adaptin-antibody-ap6-ab2730.html<br><br>mouse anti-MUC1/episialin antibody (clone 214D4, Sigma-Aldrich, #05-652): https://www.sigmaaldrich.com/US/en/product/mm/05652<br><br>rabbit anti-GFP polyclonal antibody (Sigma-Aldrich, #PC408): https://www.sigmaaldrich.com/US/en/product/mm/pc408<br><br>goat anti-mouse IgG Alexa Fluor 488 (Invitrogen, #A-11029): https://www.thermofisher.com/antibody/product/Goat-anti-Mouse-IgG-H-L-Highly-Cross-Adsorbed-Secondary-Antibody-Polyclonal/A-11029<br><br>goat anti-mouse IgG Alexa Fluor 594 (Invitrogen, #A-11032): https://www.thermofisher.com/antibody/product/Goat-anti-Mouse-IgG-H-L-Highly-Cross-Adsorbed-Secondary-Antibody-Polyclonal/A-11032

goat anti-mouse IgG Alexa Fluor 647 (Invitrogen, #A-21236): https://www.thermofisher.com/antibody/product/Goat-anti-Mouse-IgG-H-L-Highly-Cross-Adsorbed-Secondary-Antibody-Polyclonal/A-21236

goat anti-rabbit IgG Alexa Fluor Plus 647 (Invitrogen, #A-32733): https://www.thermofisher.com/antibody/product/Goat-anti-Rabbit-IgG-H-L-Highly-Cross-Adsorbed-Secondary-Antibody-Polyclonal/A32733

Eukaryotic cell lines

Policy information about cell lines

Cell line source(s)	HeLa human adenocarcinoma epithelial cells (ATCC, CCL-2™)<br>U2OS human bone osteosarcoma epithelial cells (ATCC, cat. no. HTB-96)
Authentication	Cell morphologies were observed to verify the cell line. U2OS cells were recently purchased from ATCC and the passage were carefully documented.
Mycoplasma contamination	U2OS and HeLa cells were tested and negative of mycoplasma.
Commonly misidentified lines<br>(See ICLAC register)	No commonly misidentified cells lines were used in this work.

Flow Cytometry

Plots

Confirm that:

☒ The axis labels state the marker and fluorochrome used (e.g. CD4-FITC).

☒ The axis scales are clearly visible. Include numbers along axes only for bottom left plot of group (a 'group' is an analysis of identical markers).

☒ All plots are contour plots with outliers or pseudocolor plots.

☒ A numerical value for number of cells or percentage (with statistics) is provided.

Methodology

Sample preparation	MUC1-ΔCT-expressing U2OS cells were detached non-enzymatically with a cell dissociation buffer (Gibco, #13151014) at 37? for 15-20 min. After three gentle washes with an ice-cold FACS buffer (0.5% BSA in 1X PBS), cells were stained with rabbit anti-GFP antibody (Sigma-Aldrich, #PC408) at 1:500 dilution in the FACS buffer for 30 min on ice. After washes with the ice-cold FACS buffer, cells were then labeled with goat anti-rabbit IgG Alexa Fluor Plus 647 (Invitrogen, #A32733) at 1:1000 dilution for 30 min on ice in the dark. Free antibodies were then removed by an ice-cold 2 mM EDTA in the FACS buffer. Sytox Blue (Invitrogen, #S34857) was then added to cells to check cell viability. A MACSQuant flow cytometer (Miltenyi Biotec) was used for the analysis. The raw data were further processed using FCS Express™ 7 (De Novo Software)
Instrument	MACSQuant flow cytometer
Software	FCS Express 7 Research
Cell population abundance	~3500-18000 cells per population
Gating strategy	Grating strategies used in Flow Cytometry are shown in the Supplementary Figure 1A.<br>1. Cell debris were first excluded according to the FSC-A and SSC-A,<br>2. Single cells were then gated based on FSC-A and FSC-H, and finally,<br>3. Cell viabilities were gated based on Sytox Blue staining.

☒ Tick this box to confirm that a figure exemplifying the gating strategy is provided in the Supplementary Information.
